# The emerging role of lactate in skeletal homeostasis and disorders: Integrated mechanisms and translational opportunities

**DOI:** 10.1016/j.jot.2026.101144

**Published:** 2026-06-06

**Authors:** Boyi Zong, Fengzhi Yu, Shichang Li, Peng Sun

**Affiliations:** a School of Sport Sciences, Nanjing Normal University, Nanjing, 210046, China; b School of Exercise and Health, Shanghai University of Sport, Shanghai, 200438, China; c College of Physical Education and Health, East China Normal University, Shanghai, 200241, China; d Key Laboratory of Adolescent Health Assessment and Exercise Intervention of Ministry of Education, East China Normal University, Shanghai, 200241, China

**Keywords:** Lactate metabolism, Lactate receptor, Lactate shuttle, Lactylation, Skeletal disorders, Skeletal system

## Abstract

Lactate has evolved from a metabolic byproduct to a central regulator of skeletal homeostasis and disorders, integrating metabolic flux with epigenetic reprogramming. Within the skeletal system, it modulates the processes of osteogenesis, osteoclastogenesis, chondrogenesis, and matrix synthesis by regulating the physiological activities of bone marrow mesenchymal stem cells, osteoblasts, osteoclasts, chondrocytes, and intervertebral disc cells, thereby maintaining skeletal homeostasis. Dysregulated lactate signaling is increasingly recognized as a pivotal pathological mechanism in degenerative, inflammatory, and neoplastic skeletal disorders. The presence of pathogenic lactate flux has been shown to drive disease-specific mechanisms, including, in osteoporosis, it disrupts osteoblast-osteoclast coupling; in osteoarthritis, it amplifies chondrocyte inflammation and matrix degradation; in rheumatoid arthritis, excess lactate enhances fibroblast invasiveness; in intervertebral disc degeneration, lactate accumulation induces acidosis and cell death; and in malignancies, lactate simultaneously nourishes osteoclasts and suppresses anti-tumor immunity. Currently, emerging preclinical evidence indicates that therapeutic strategies targeting lactate metabolism, transport, receptor signaling, and lactylation show promise. However, challenges such as cell-type-specific effects, metabolic compensation, and systemic off-target risks persist.

**The translational potential of this article:**

This review comprehensively examines the regulatory role and mechanisms of lactate in skeletal homeostasis and evaluates the therapeutic potential of lactate-based interventions for skeletal disorders. The advancement of lactate-based interventions is contingent on the development of bone-specific delivery systems and personalized approaches informed by metabolic-epigenetic profiles, offering promising new avenues for treating skeletal disorders.

## Graphical abstract

This review delineates the central role of lactate in skeletal physiology and pathology. It examines how lactate regulates the function of bone marrow mesenchymal stem cells, osteoblasts, osteoclasts, chondrocytes, and intervertebral disc cells to maintain tissue homeostasis. Furthermore, the study elucidates its pathogenic involvement in disorders including osteoporosis, osteoarthritis, rheumatoid arthritis, intervertebral disc degeneration, and osteosarcoma, thereby highlighting its potential as a therapeutic target for skeletal disorders.

**Figure:**
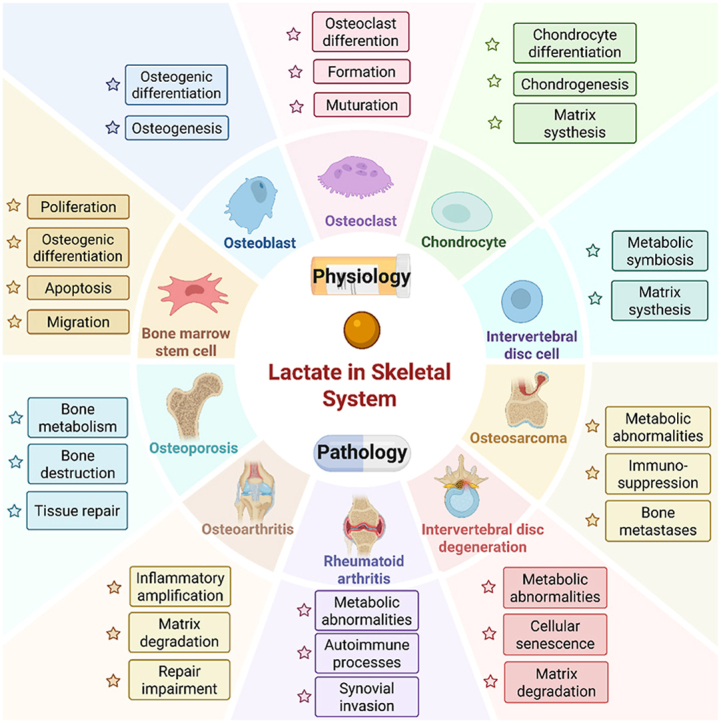

## 1 Introduction

Lactic acid is one of the most abundant circulating metabolites in the human body. Under normal physiological pH conditions, the substance exists primarily as the lactate anion, formed through the reduction of the end product of glycolysis, pyruvate, by lactate dehydrogenase (LDH) [1]. Traditionally regarded as the end product of anaerobic metabolism, this perception has undergone a fundamental revolution. Subsequent to the formulation of the “lactate shuttling” hypothesis, the role of lactate as a pivotal energy substrate and gluconeogenic precursor has been firmly established [2]. This compound is actively transported between various tissues (e.g., muscle, liver, and heart) via monocarboxylate transporters (MCTs) [2]. Its role in coordinating energy metabolism and maintaining homeostasis during exercise physiology has also been confirmed [3]. Recent research has further expanded the boundaries of its biological significance, revealing that lactate exerts extensive pathophysiological regulatory functions across multiple systems, including cardiovascular dynamics [4] and immune modulation [5]. The comprehensive functional repertoire has profoundly transformed the conceptualization of lactate, transitioning it from a mere metabolic endpoint to a dynamic pleiotropic mediator.

**Fig. 1:**
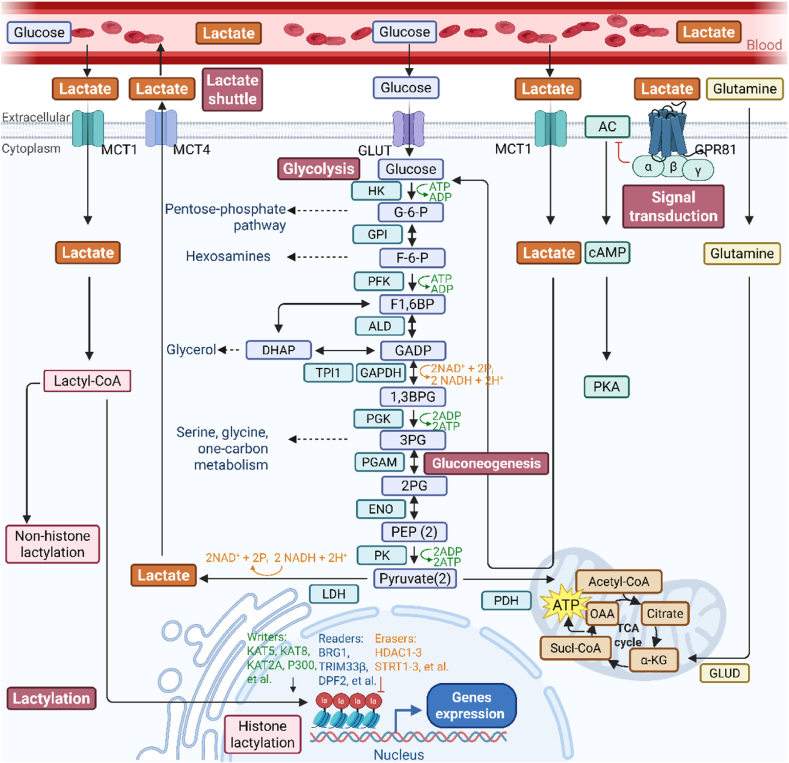
Schematic illustration of lactate metabolism, transport, sensing, and lactylation in cells. In the cytoplasm, lactate is transported into cells by MCT1 and is primarily produced from glycolysis. The catabolism of lactate in cells occurs through two distinct mechanisms. On the one hand, lactate is oxidized to pyruvate, which enters the mitochondria and is metabolized through the TCA cycle. On the other hand, lactate is converted to glucose through the process of gluconeogenesis. Lactate can be converted into lactyl-CoA, and it has been demonstrated to be involved in the lactylation of histones and non-histone proteins. Furthermore, lactate can be transported out of cells by MCT4 and enter the circulatory system. Extracellular lactate has been demonstrated to activate GPR81, thus regulating the activity of signaling pathways such as AC/cAMP/PKA. **Abbreviations:** 1,3BPG, 1,3-Bisphoshoglycerate; 3 PG, 3-Phosphoglycerate; 2 PG, 2-Phosphoglycerate; α-KG, α-Ketoglutarate; AC, adenylate cyclase; ADP, adenosine diphosphate; Acetyl-CoA, Acetyl Coenzyme A; ALD, aldolase; BRG1, Brahma-related gene 1; DHAP, dihydroxyacetone phosphate; DPF2, dual PHD-D4 zinc finger protein 2; ENO, enolase; F1,6BP, fructose-1, 6-phosphate‌; F-6-P, fructose-6-phosphate; G-6-P, glucose-6-phosphate; GADP, glyceraldehyde 3-phosphate; GAPDH, glyceraldehyde-3-phosphate dehydrogenase; GLUD, glutamate dehydrogenase; GLUT, glucose transporter; GPI, glucose phosphate isomerase; HK, hexokinase; KAT, lysine acetyltransferase; OAA, oxaloacetic acid; PDH, pyruvate dehydrogenase; PEP, phosphoenolpyruvate; PFK, phosphofructokinase; PGK, phosphoglycerate kinase; PGAM, phosphoglycerate mutase; PK, pyruvate kinase; PKA, protein kinase A; Sucl-CoA, Succinyl-CoA; TPI1, triosephosphate isomerase 1; TRIM33β, tripartite motif-containing 33β; ↑, activation or upregulation; ⊥, inhibition or downregulation. The figure was created using the BioRender.

This functional versatility is underpinned by its intricate biochemistry and signaling modalities. At the metabolic level, the synthetic reaction catalyzed by lactate dehydrogenase A (LDHA) constitutes a vital pathway for maintaining glycolytic flux and rapid adenosine triphosphate (ATP) regeneration, a principle observed in intensely contracting muscles and tumor cells exhibiting the Warburg effect [6,7]. Conversely, the oxidative reaction catalyzed by lactate dehydrogenase B (LDHB) facilitates the conversion of lactate into glucose or other biosynthetic precursors within tissues such as the liver [8,9]. The lactate shuttle mechanism, facilitated by cell-type-specific MCT isoforms (e.g., MCT1 for uptake, MCT4 for export), enables this metabolite to function as a crucial energy currency and gluconeogenic precursor across tissues and organs. This process is regulated by factors like hypoxia-inducible factor 1α (HIF-1α) and peroxisome proliferator-activated receptor γ coactivator 1α (PGC-1α) [10,11]. Beyond its metabolic roles, lactate has been shown to act as a signaling molecule via its specific receptor, G protein-coupled receptor 81 (GPR81). The activation of this receptor has been demonstrated to modulate intracellular cyclic adenosine monophosphate (cAMP) levels and engage β-arrestin-mediated pathways [12,13]. Most recently, lactate has been identified as the source for a novel form of post-translational modification, lysine lactylation, which regulates gene expression and protein function by modifying histones and non-histone proteins, adding a direct epigenetic and regulatory layer to its biological function [14,15]. It is imperative to acknowledge that the process of lactylation is a dynamic and reversible process involving a series of enzyme-catalyzed reactions. The enzymes responsible for the addition of lactyl groups to proteins are termed “Writers” and include General Control Non-derepressible 5-related N-acetyltransferase (GNAT), MOZ, Ybf2/Sas3, Sas2, and Tip60 (MYST) histone acetyltransferase‌, and p300/cAMP-response element binding protein (CREB)-binding protein‌ (CBP), among others. Delactylases, also known as “Erasers”, are primarily comprised of histone deacetylase 1-3 (HDAC1-3) and silent mating type information regulation 2 homolog 1-3 (SIRT1-3). The enzymes containing the Bromodomain (BRD) domain, Plant Homeodomain (PHD) zinc finger domain, and Yaf9, ENL, AF9, Taf14, Sas5 (YEATS) domain function as “Readers”, recognizing and binding to acetylated histones to regulate chromatin structure and gene expression [16] (Fig. 1).

The skeletal system constitutes 15-20% of body mass, providing essential structural support and organ protection [17], as well as housing marrow for hematopoiesis and immunity, thus establishing an integrative physiological hub [18]. Additionally, it functions as a dynamic metabolic and endocrine organ, regulating systemic homeostasis [19]. The coordinated activities of various cell types are required for the sustained functioning of the skeletal system. This ecosystem principally comprises bone mesenchymal stem cells (BMSCs), which are located in the bone marrow and function as osteochondroprogenitors and stromal components, as well as bone-forming osteoblasts (OBs), bone-resorbing osteoclasts (OCs), cartilage-producing chondrocytes, and specialized cells within the intervertebral discs (IVD). The functional synergy between these cells is essential for maintaining skeletal structural integrity, mineral homeostasis and metabolic fitness [20]. Emerging evidence indicates that the signaling of lactate, encompassing its metabolism, shuttle (via MCTs), sensing (via GPR81), and lactylation, functions as a central regulator of skeletal physiology. This regulatory system has been implicated in major skeletal pathologies, including osteoporosis (OP), osteoarthritis (OA), rheumatoid arthritis (RA), intervertebral disc degeneration (IVDD), and bone malignancies [[21], [22], [23], [24], [25]]. In light of these advances, the present integrative review aims to synthesize current knowledge on the role of lactate in the physiology and pathology of the skeletal system. In order to ensure a comprehensive and systematic synthesis, a search was conducted for literature published up to March 2026 from the PubMed and Web of Science databases. This was achieved using keyword combinations of core terms (e.g., “lactate”, “lactic acid”) and skeletal system components (e.g., “bone”, “cartilage”, “osteoporosis”, “osteoarthritis”). A manual screening of the reference lists of key articles was conducted to identify additional relevant studies. The selection of studies was prioritized in accordance with human clinical and translational research, followed by animal studies that provide mechanistic insights and in vitro findings that elucidate fundamental cellular pathways. In instances where contradictory evidence has been identified, both perspectives are presented and discussed within relevant thematic sections in order to provide a balanced and critical analysis. This review aims to elucidate the application of targeting lactate regulation to control skeletal homeostasis and treat skeletal disorders.

## 2 Lactate biosynthesis and multifunctional roles in the skeletal cellular microenvironment

**Fig. 2:**
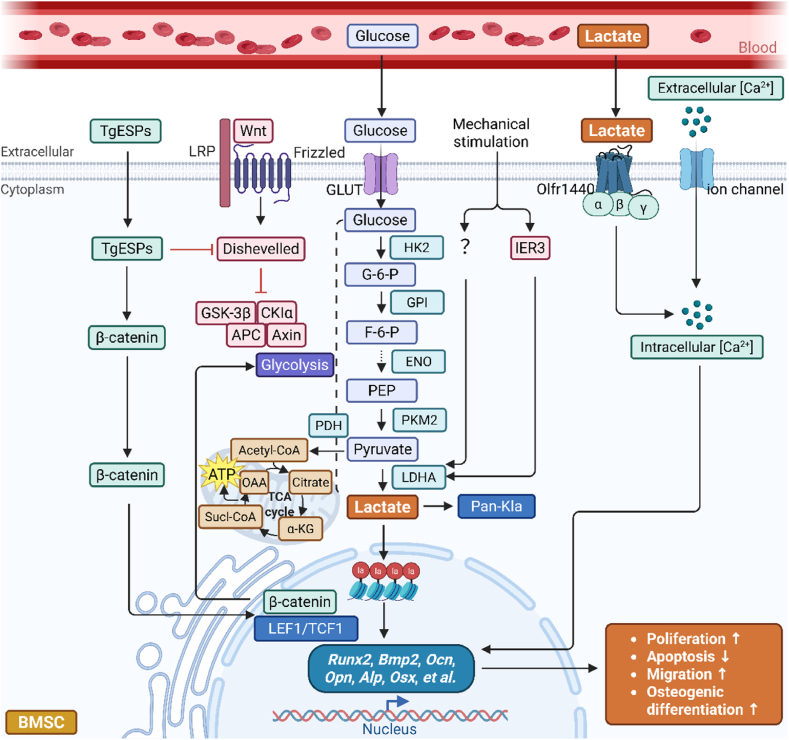
Schematic illustration of the roles of lactate in BMSC. In BMSC, it has been demonstrated that exogenous stimuli, including chemical signals (e.g., TgESPs) and mechanical cues, have the capacity to modulate glycolytic activity, thereby altering lactate production. This, in turn, influences the proliferation, differentiation, migration, and apoptosis of BMSCs through metabolic-epigenetic crosstalk. Concurrently, lactate has been demonstrated to activate the Olfr1440 and its downstream Ca^2+^ signaling axis. **Abbreviations:** APC, Axin-containing protein complex; ALP, alkaline phosphatase; CKIα, casein kinase Iα; LEF, Leucine-rich repeat-containing G-protein coupled receptor; OPN, osteopontin; OSX, Osterix; TCF, transcriptional co-activator/leucine zipper factor.

**Fig. 3:**
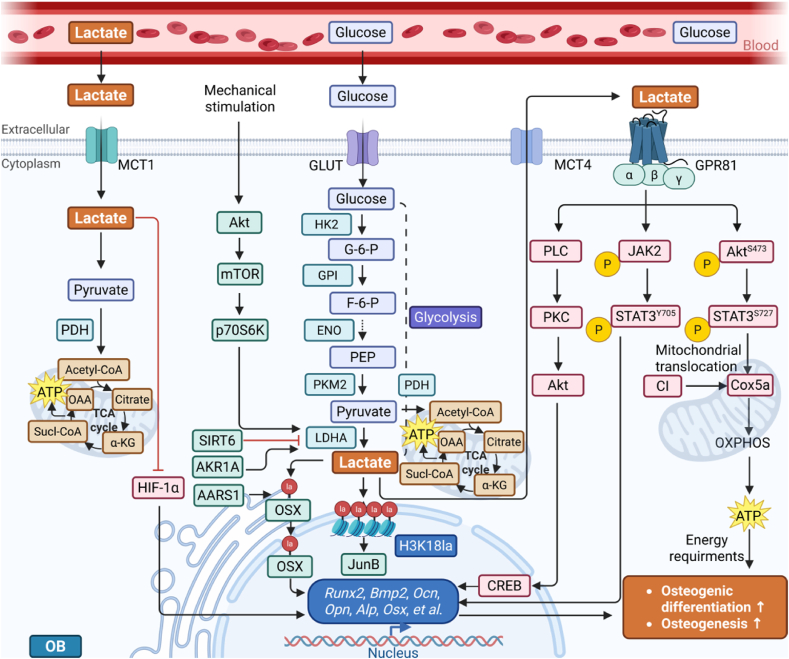
Schematic illustration of the roles of lactate in OB. In OBs, mechanical signals, specific signaling molecules such as Wnt, and enzymes like AKR1A have been shown to influence lactate production in OBs by affecting glycolytic processes. Lactate has been demonstrated to promote OXPHOS and inhibit HIF-1α activity, while also acting on osteogenic gene expression by mediating lactylation levels, such as H3K18la and OSX lactylation. Furthermore, lactate has been demonstrated to modulate the process of osteogenic differentiation through the activation of GPR81 and its associated downstream signaling pathways, including PLC/PKC/Akt/CREB, JAK2/STAT3, and Akt/STAT3, among others. **Abbreviations:** CI, complex I; Cox5a, cytochrome c oxidase subunit Va‌; PKM2, pyruvate kinase isozymes M2.

**Fig. 4:**
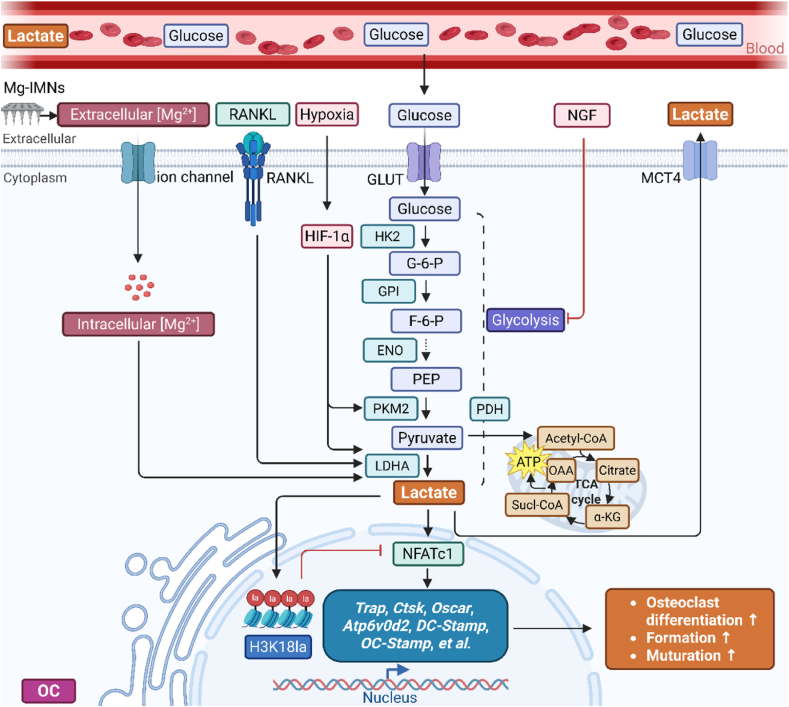
Schematic illustration of the roles of lactate in OC. In OC, it has been demonstrated that HIF-1α, RANKL signaling, and NCF can regulate glycolytic enzyme activity and affect lactate production. Lactate has been observed to exert a bidirectional regulatory effect. On the one hand, lactate has been observed to promote the formation of OCs and the process of bone resorption, as well as increasing the expression of NFATc1. However, Mg^2+^ has been demonstrated to induce the production of intracellular lactate, which subsequently results in histone lactylation. This, in turn, has been demonstrated to suppress OC maturation by means of downregulating NFATc1. The p300/H3K18la/HDAC1 pathway has been identified as a key mediator in this process. **Abbreviations:** ATP6V0d2, ATPase, H^+^ transporting, lysosomal 38 kDa, V0 subunit d2; CTSK, cathepsin K; DC-STAMP, dendrocyte expressed seven transmembrane protein; OC-STAMP, OC stimulatory transmembrane protein; OSCAR, OC-associated receptor; TRAP, tartrate-resistant acid phosphatase; ↑, activation or upregulation; ↓, inhibition or downregulation.

**Fig. 5:**
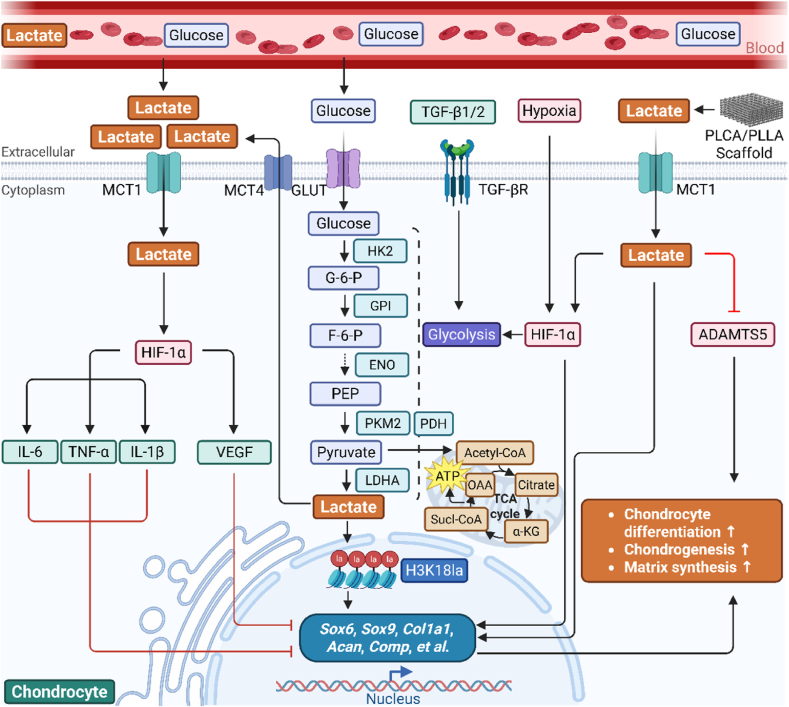
Schematic illustration of the roles of lactate in chondrocyte. In chondrocyte, inflammation and aerobic glycolysis form a positive feedback loop. Exposure to inflammatory factors has been demonstrated to promote lactate production by inducing aerobic glycolysis. It has been demonstrated that excessive lactate accumulation enhances inflammatory factor secretion via the HIF-1α pathway. Furthermore, the appropriate dose of lactate has been shown to inhibit the expression of cartilage matrix degradation genes in chondrocytes, such as ADAMTS5, whilst concomitantly promoting matrix synthesis. It has been demonstrated that lactate exerts a direct effect on H3K18la. The process of enhanced lactylation has been demonstrated to promote the formation of cartilage matrix by improving the accessibility of genes involved in the process of cartilage formation. **Abbreviations:** ACAN, aggrecan; ADAMTS5, a disintegrin and metalloproteinase with thrombospondin motifs 5; SOX, SRY-Box transcription factor.

**Fig. 6:**
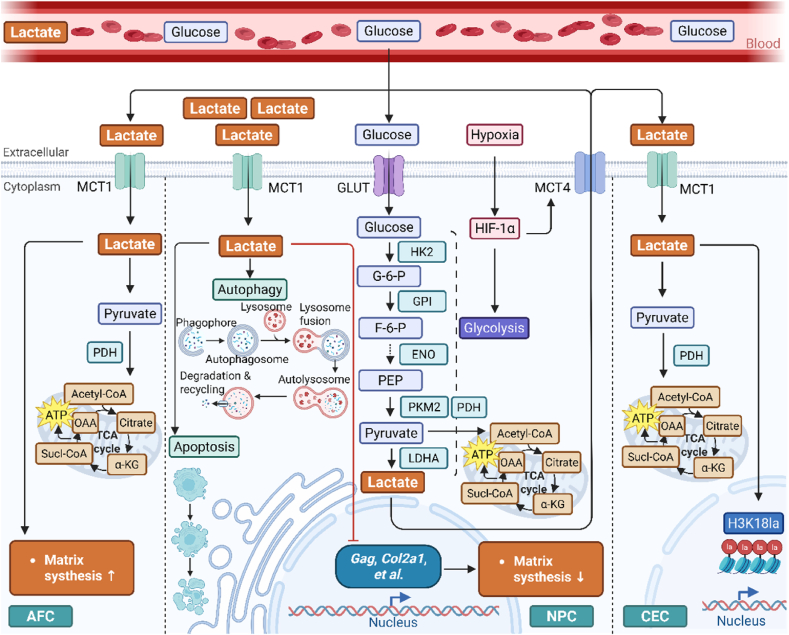
Schematic illustration of the roles of lactate in IVD cells. In NPCs, lactate produced by glycolysis may be transported to AFCs and CECs, thereby promoting ATP production to meet energy demands. Moreover, in NPCs, excessive lactate has been shown to induce apoptosis and autophagy, whilst concurrently inhibiting matrix synthesis. In AFCs, lactate has been observed to promote matrix synthesis. In CECs, lactate has been shown to induce an increase in H3K18la levels. **Abbreviations:** CECs, cartilage endplate cells.

The maintenance of skeletal integrity is contingent upon the synergy of cells within a dynamic ecosystem, wherein lactate functions as a pivotal metabolic signaling molecule. As demonstrated in Fig. 2, Fig. 3, Fig. 4, Fig. 5, Fig. 6, lactate has been shown to exert a regulatory influence on a number of processes, including bone formation, bone resorption, cartilage formation and matrix synthesis.

### 2.1 Lactate in bone marrow mesenchymal stem cells

Osteoprogenitor cells have been demonstrated to exhibit dynamic regulatory functions in relation to lactate production, in response to a variety of physiological and pathological stimuli. While chronic intermittent hypoxia has been demonstrated to promote lactate accumulation, low glucose availability has been shown to suppress it, reflecting metabolic adaptation to the local microenvironment [26,27]. Mechanical signals have been demonstrated to represent a potent anabolic stimulus, whereby mechanical loading or exercise enhances LDH activity, thereby driving lactate synthesis via proteins such as immediate early response 3 (IER3) and concomitant expression of osteogenic and proliferative markers in BMSCs [[28], [29], [30]]. Furthermore, molecular cues such as those derived from the protozoan Toxoplasma gondii have also been shown to interfere with this metabolic programme, stimulating glycolysis and osteogenic differentiation in BMSCs through Wingless-related integration site (Wnt)/β-catenin signaling [31]. The pivotal function of Wnt signaling is highlighted by the observation that Wnt3a, via low-density lipoprotein receptor-related protein 5 (LRP5), activates a Ras-related C3 botulinum toxin substrate 1 (RAC1)/mammalian target of rapamycin (mTOR) complex 2 (mTORC2)/protein kinase B (PKB, i.e. Akt) cascade to upregulate LDHA, elevate circulating lactate, and promote osteogenesis [32].

Lactate itself functions as a critical feedback signal, exerting context-dependent effects on osteogenesis. It has been demonstrated that exogenous lactate has the capacity to shift MSC metabolism towards oxidative phosphorylation (OXPHOS) and to enhance glutamine utilization, thereby supporting a more differentiated state [33]. During the process of fracture repair, a local surge in lactate activates the olfactory receptor 1440 (Olfr1440), thereby triggering an influx of calcium and upregulating osteogenic genes such as Runt-related transcription factor 2 (Runx2), bone morphogenetic protein 2 (BMP2), and osteocalcin (OCN), enhancing the regenerative capacity of BMSCs, and promoting the healing process [34] (Fig. 2). Conversely, there is evidence to suggest that chronic, intermittent hypoxia-induced long-term elevated lactate levels may have an adverse effect on skeletal development [27]. Hypoxia-driven lactate accumulation has been observed to enhance histone H3 lysine 18 lactylation (H3K18la) at the peroxisome proliferator-activated receptor γ (PPARγ) promoter in BMSCs, activating this adipogenic master regulator. This, in turn, has been shown to skew lineage allocation towards adipocytes, thereby disrupting long bone growth [27]. In BMSCs, it has been observed that the production of lactate is subject to precise regulation in response to various microenvironmental signals, including conditions of hypoxia and mechanical loading. Concurrently, lactate exerts divergent effects on osteogenic differentiation: in specific physiological scenarios such as fracture repair, it promotes bone formation by activating receptors such as Olfr1440; whereas in pathological conditions such as chronic hypoxia, its sustained accumulation can activate adipogenic programmes by inducing epigenetic modifications such as H3K18la, thereby impairing skeletal development.

### 2.2 Lactate in osteoblast and osteocyte

A substantial body of research suggests that mechanical stimulation and specific signaling pathways collectively constitute the core mechanism regulating the activity of the glycolysis and lactate production of the OB. At the mechanosensory interface, cyclic stretching directly enhances glycolytic flux and lactate output in OB-like cells via activation of the Akt/mTOR/70 kDa ribosomal protein S6 kinase (p70S6K) pathway [35]. In addition, canonical Wnt/β-catenin signaling has been demonstrated to orchestrate a transcriptional metabolic switch. Upon pathway activation, stabilized β-catenin translocates to the nucleus and binds the promoter of pyruvate dehydrogenase kinase isozyme 1 (PDK1), upregulating its expression to divert pyruvate from mitochondrial oxidation toward lactate generation [36]. The Wnt co-receptor leucine-rich repeat-containing G protein-coupled receptor 4 (LGR4) has been demonstrated to play a pivotal role in regulating this axis. Its absence has been shown to suppress glucose uptake, lactate production, and PDK1 expression. This phenotype can be reversed by β-catenin expression, thereby confirming the LGR4-Wnt-PDK1 axis as a core metabolic regulator [36]. Furthermore, the intracellular redox state, governed by factors such as the nicotinamide adenine dinucleotide phosphate (NADPH)-dependent aldehyde reductase aldehyde reductase family 1 member A (AKR1A), is deeply intertwined with OB metabolism. AKR1A has been shown to regulate the NADP^+^/NADPH redox couple, nitric oxide levels, glucose uptake, and lactate production [37]. Concurrently, it exerts influence on the expression of the collagen type I alpha 1 (COL1A1), receptor activator of nuclear factor κB (NF-κB) ligand (RANKL) and osteoprotegerin (OPG). This finding indicates a potential relationship between cellular redox homeostasis and the anabolic and catabolic processes involved in bone signaling. Collectively, these inputs from mechanotransduction, lineage-defining pathways, and redox balance converge to precisely configure the bioenergetic state of OBs, thereby underpinning their bone-forming function.

Recent studies have revealed the critical, stage-specific role of lactate metabolism in the differentiation and function of OBs and osteocytes. During the late stages of OB-to-osteocyte differentiation, cells undergo significant metabolic reprogramming, characterized by increased glucose consumption, upregulation of key glycolytic enzymes (e.g., PKM and LDHA), and a surge in lactate production [38]. This enhancement in glycolytic flux coincides with the onset of osteocyte maturation marker expression. Furthermore, the inhibition of glucose metabolism at this particular stage has been shown to impede terminal differentiation and matrix mineralization [38], suggesting a pivotal role for glycolysis and lactate in the process of osteocyte differentiation. In contrast, mature osteocytes have been shown to exhibit metabolic flexibility. Short-term inhibition of glycolysis has been demonstrated to downregulate the Wnt pathway inhibitor sclerostin (SOST) and the phosphoregulatory factor fibroblast growth factor 23 (FGF23), while concomitantly upregulating genes associated with fatty acid β-oxidation [38]. This finding indicates that post-differentiated osteocytes possess the capacity to transition between different energy substrates, and that an oxidative metabolic state may be a hallmark of the osteogenic phenotype. This metabolic flexibility is also linked to mechanical responses; for instance, mechanical loading (e.g., fluid shear stress) upregulates genes associated with oxidative metabolism and fatty acid β-oxidation [38]. In addition to its role as a metabolite, lactate is also a key signaling molecule that regulates osteocyte function. Extracellular acidosis has been demonstrated to significantly promote the transcription of SOST and OPG in osteocytes [39]. This process is thought to involve acidosis-mediated activation of calcium/calmodulin-dependent kinase II (CaMKII) signaling, which reduces the nuclear localization of HDAC5, thereby alleviating its inhibition on SOST [39]. Consequently, lactate and acidosis can directly regulate osteocyte function, influencing the key regulators of bone formation and resorption.

In addition, with regard to the function of lactate in OB differentiation, at low concentrations, the lactate has a promoting effect, whereas at high concentrations, it has an inhibitory effect [40]. Lactate has been demonstrated to influx through MCT1, leading to a reduction in HIF-1α activity and an increase in ALP activity and OCN expression [40]. In addition, lactate activates GPR81, triggering signaling cascades involving Gβγ, phospholipase C (PLC), protein kinase C (PKC), Akt and Janus kinase 2 (JAK2)/signal transducer and activator of transcription 3 (STAT3) [41,42]. These cascades have been demonstrated to enhance osteogenic marker expression and mitochondrial biogenesis. Epigenetically, lactylation (e.g., H3K18la) have been demonstrated to further enhance transcriptional activity at key osteogenic gene loci such as COL1A2, cartilage oligomeric matrix protein (COMP), and Jun B proto-oncogene (JunB) [21,43,44]. Furthermore, lactate has been demonstrated to directly lactylate the STAT1, thereby promoting its release and subsequent activation of Runx2 [45]. A parallel mechanism involves alanyl-tRNA synthetase 1 (AARS1), which catalyzes the lactylation of OSX, enhancing its DNA-binding affinity and interaction with the epigenetic regulator WD repeat domain 5 (WDR5) to drive H3K4 trimethylation and target gene expression [46]. However, in pathological contexts, the metabolic process of lactate becomes detrimental to the individual. Downregulation of the deacetylase SIRT6 has been demonstrated to exacerbate glycolysis and lactate accumulation, thereby synergistically increasing reactive oxygen species (ROS) and pro-inflammatory factors [[47], [48], [49]]. These factors have been shown to drive OB apoptosis and dysfunction. Within an acidic bone microenvironment, excess lactate has also been demonstrated to impede angiogenesis by suppressing vascular endothelial growth factor (VEGF) expression and to promote pathological calcification via B cell lymphoma 2 (Bcl2) adenovirus E1B 19 kDa interacting protein 3 (BNIP3)-dependent disruption of mitophagy [[50], [51], [52], [53], [54]] (Fig. 3). In OBs and osteocytes, the presence of lactate has been demonstrated to stimulate osteogenesis, operating through the mediation of receptors such as GPR81 and by means of lactate modification. Conversely, there is evidence to suggest that pathological overaccumulation of lactate can drive oxidative stress and inflammation, thereby impairing bone formation.

### 2.3 Lactate in osteoclast

The process of OC differentiation is driven by a significant reorganization of cellular metabolism, with aerobic glycolysis providing a fundamental energetic and biosynthetic foundation. The metabolic programme is subject to dynamic regulation by the bone microenvironment. The hypoxic marrow niche, through the action of HIF-1α, has been demonstrated to upregulate the expression of LDHA, PKM2 and PFK1, with the objective of enhancing glycolytic flux and lactate output. This reprogramming is further tuned by the 6-phosphofructo-2-kinase/fructose-2,6-bisphosphatase 3 (PFKFB3), whose inhibition curtails lactate accumulation and impairs osteoclastogenesis. Furthermore, the process is also initiated by the RANKL, which orchestrates a coordinated surge in LDH activity, glycolytic rate, and mitochondrial respiration [[55], [56], [57]] (Fig. 4). The synchronized flux of glycolytic-lactate metabolism and OXPHOS powers OCs while fueling the auto-amplifying, nuclear factor of activated T-cells cytoplasmic 1 (NFATc1)-driven transcriptional programme that dictates cell fusion and resorptive function. The disruption of this metabolic pathway, whether through the genetic ablation of *Ldha* or *Ldhb* or the pharmacological inhibition of LDHA with FX11, results in the incapacitation of both energy pathways, NFATc1 signaling, and bone resorption [58,59]. The nuciferine (NGF) has been demonstrated to reliably inhibit osteoclastogenesis through the attenuation of hexokinase 2 (HK2), PKM2, and LDHA expression, accompanied by a reduction in lactate production. In contrast, the activation of PKM2 or the addition of lactate has been observed to promote differentiation and resorptive gene expression [60] (Fig. 4).

The spatial control of lactate, governed by specialized MCTs, adds another layer of regulation. Myeloid cells exhibit a striking MCT isoform switch: macrophages primarily utilize MCT1 and MCT4, whereas mature OCs shift dominance to MCT2. In essence, MCT1 exerts a regulatory effect on the process of differentiation, while MCT2 is indispensable for the process of bone resorption. This provides a scientific rationale for the biphasic effect of broad MCT inhibition [61]. Furthermore, lactate has been demonstrated to exert a direct influence on the OC lineage through epigenetic mechanisms. In the process of fracture healing, magnesium-induced lactate production has been shown to trigger H3K18la [62]. This, in turn, has been found to recruit HDAC1, thereby suppressing NFATc1 transcription via a p300-dependent pathway. Consequently, this process has been demonstrated to inhibit the maturation of OCs. In summary, the reprogramming of glycolysis triggered by RANKL and hypoxia signals provides the essential energy and biosynthetic precursors required for OC differentiation. Key proteins such as LDHA and MCT1/2 have been demonstrated to facilitate the production and transport of lactate, thereby supporting the differentiation process and the resorption function of mature cells, respectively. Furthermore, lactate has been demonstrated to instigate a feedback regulatory loop by inducing H3K18la.

### 2.4 Lactate in chondrocytes

Articular cartilage, being avascular, exhibits a stratified functional architecture defined by its nutrient and oxygen supply. Nutrients are primarily supplied to the joint via diffusion from the synovial fluid, creating a steep physiological gradient: the superficial zone is relatively oxygenated, while the deep zone is profoundly hypoxic. Chondrocytes in superficial layers predominantly rely on OXPHOS for energy production. In contrast, those residing in the middle and deep zones, confronted with limited oxygen availability due to hypoxia, increasingly resort to anaerobic glycolysis as a primary source of energy, a process inherently linked to lactate production [[63], [64], [65], [66]]. In conditions of hypoxia, the stabilization and sustained activation of HIF-1α has been demonstrated to upregulate glucose transporter 1 (GLUT1) and LDHA expression in chondrocytes, thereby promoting lactate production [67,68], while the knockdown of *Pkm2* has been shown to result in a significant inhibition of glucose consumption, lactate production, and GLUT1, LDHA, and HIF-1α expression [69]. Moreover, it has been established that several molecular pathways, including transforming growth factor β (TGF-β), play a critical role in maintaining chondrocyte homoeostasis. In particular, the protein known as TGF-β1 increases key regulators of glycolysis (e.g., GLUT1 and HK2), thus stimulating the process of glycolysis and increasing lactate production [70]. TGF-β2 signaling has been demonstrated to upregulate ATP-dependent 6-PFK expression and promote lactate production via the TGF-β receptor type I (TβRI) and phosphorylated Sma and Mad​ related protein 3 (Smad3) signaling pathway [71]. Furthermore, a positive feedback loop is established between inflammation and aerobic glycolysis in chondrocytes. Exposure to proinflammatory cytokines such as interleukin 6 (IL-6) and tumor necrosis factor α (TNF-α) have been shown to induce aerobic glycolytic reprogramming in cartilage tissue and to upregulate LDHA expression by activating the canonical NF-κB signaling pathway [72]. This, in turn, establishes a positive feedback loop between inflammation and metabolism. In mouse chondrocytes line ATDC5, which were exposed to IL-1β, an increase in lactate production was observed prior to the onset of cell death. The suppression of IL-1β-induced intracellular ROS production and cell death was effectively achieved through the knockdown of the *Mct1* gene [73]. Furthermore, enhanced MCT4 activity has been demonstrated to promote lactate clearance from chondrocytes, thereby reducing intracellular pH and influencing cartilage renewal [74]. It has been demonstrated that excessive extracellular lactate has the capacity to stabilize HIF-1α protein, thus inducing the expression of VEGF and pro-inflammatory factors [75] (Fig. 5). This process is known to promote glycolysis. Consequently, the production of lactate within these cells is influenced by a range of factors, including hypoxia and inflammation, as well as specific molecular pathways, such as those involving the TGF-β family. The transport of lactate in chondrocytes is subject to strict regulation by MCT.

The effects of lactate on chondrocyte matrix synthesis exhibit time- and dose-dependent characteristics. It has been demonstrated that chronic lactate exposure (100 mM, 8h) and pulsed addition of lactate both have the capacity to promote matrix synthesis. However, the resultant acidic pH, consequent to excessive lactate accumulation, has been demonstrated to inhibit the proliferation of chondrocytes and the synthesis of the cartilage matrix [76]. In accordance with this, in a cobalt chloride-simulated hypoxia model, the upregulation of matrix metalloproteinase 2 (MMP-2) alongside lactate accumulation has been demonstrated to exert detrimental effects on the extracellular matrix (ECM) [64]. At the epigenetic level, enhanced H3K18la has been demonstrated to promote the process of chondrogenesis and cartilage matrix deposition by improving the accessibility of the genes responsible for this process (Fig. 5). The implantation of the ultradynamic hydrogel in large animal cartilage defects has been shown to result in superior repair compared to less dynamic alternatives [77], thus providing a valuable source of insights for effective biomaterial delivery in cell therapies.

### 2.5 Lactate in intervertebral disc cells

The IVD is a biomechanically highly integrated structure situated within an avascular, hypoxic and acidic microenvironment. The structure is composed of three distinct yet interdependent regions: the proteoglycan-rich and hydrated nucleus pulposus (NP) core, the annulus fibrosus (AF) arranged in concentric circles, and the hyaline cartilage endplates (CEPs) composed of hyaline cartilage [78,79]. In this microenvironment, a sophisticated metabolic symbiosis centered on lactate has been elucidated. In situations of severe hypoxia, NP cells (NPCs) have been observed to adopt a function as net lactate producers. This transition is accompanied by elevated expression levels of the *Ldh5* and *Mct4* genes. The HIF-1α-driven expression of solute carrier family 16, member 3 (SLC16A3), which is responsible for encoding MCT4, is critical for maintaining this efflux. Evidence suggests that *Mct4* deletion disrupts both glycolytic and mitochondrial metabolic flux [80,81]. In contrast, AF cells (AFCs) and EP cells (EPCs) are metabolic consumers. The expression of high levels of the MCT1 and LDH1 enables the cells to import NP-derived lactate and channel it into the TCA cycle for OXPHOS and biosynthetic pathways, such as collagen production [80] (Fig. 6). This metabolic state is subject to dynamic tuning in response to mechanical load. Dynamic compression has been demonstrated to stimulate glycolytic flux and lactate production in AFCs, concomitantly elevating lactate concentrations in the nutrient-depleted NP. This establishes a link between mechanical demand and metabolic output [82,83]. Conversely, the elimination of mechanical stress, as observed in microgravity, results in a shift towards reduced lactate levels in human IVD metabolism, thereby emphasizing the physiological role of load in maintaining the disc's characteristic metabolic state [84]. The extant evidence suggests that mechanical stimulation regulates IVD cell metabolism through differentiated mechanosensory mechanisms.

It has been demonstrated that lactate exerts concentration-dependent regulatory effects on the survival, metabolism, and matrix homeostasis of IVD cells. Research has indicated that a 2 mM concentration of lactate promotes the proliferation of NPCs, whereas 6 mM lactate induces autophagy and apoptosis accompanied by decreased glycosaminoglycan (GAG) content and COL2 expression [85] (Fig. 6). Furthermore, it has been determined that lysine lactylation is a pivotal regulatory element in NPC metabolism. Liquid chromatography-tandem mass spectrometry analysis of rat NPCs under normoxic and hypoxic conditions has identified 3510 lactylation sites across 1052 non-histone proteins, thereby demonstrating the breadth of this modification [86]. Bioinformatics analysis revealed a significant enrichment of lactylated proteins in ribosomal biogenesis, spliceosome dynamics and the VEGFA-VEGF receptor 2 (‌VEGFR2) signaling cascade [86]. In addition, a preprint study (which has not been certified by peer review) reported that lactate has a promoting effect on protein synthesis and induces H3K18la in EPCs [87] (Fig. 6). Within the cells of the IVD, a metabolic symbiosis of lactate-based nature is present amongst those of the NPs, the AFs and the CEPs; this is a vital element in maintaining homeostasis within the disc and adapting to mechanical loads. The effects of lactate itself are typically concentration-dependent and may exert bidirectional regulatory effects on cell survival, matrix synthesis and degradation.

## 3 Lactate as a therapeutic target in skeletal pathologies

The deterioration of the skeletal system that occurs with age is a major cause of morbidity, and this underscores the urgent need for therapeutic strategies that are informed by mechanistic understanding [88]. It has been established that lactate signaling dysregulation is a shared underlying factor in a range of skeletal pathologies. In order to elucidate the dual role of lactate in both pathogenesis and therapeutic effects, it is necessary to conduct an in-depth analysis of the regulatory roles and mechanisms of lactate metabolism, transport, sensing and lactylation in different disorders. The subsequent section delineates five major skeletal disorders: OP, OA, RA, IVDD and OS (Table 1, Table 2, Table 3, Table 4, Table 5).

**Table 1:** The potential of multiple strategies for targeting lactate and its upstream and downstream signaling in OP.

Model type and sample sizes	Treatments	Mechanism of action	Main outcomes	References
Animal Model: C57BL/6 mice, calvarial osteolysis and OVX-induced OP model. Cellular Model: Mouse BMMs.	Intervention: Visomitin. Dose & Route: In vivo: Local or systemic administration. In vitro: Applied to BMMs. Safety alert: No overt toxicity.	Visomitin directly interacts with STAT3; STAT3 activity↓; LDHB↓, Glycolytic flux↓; Lactate↓, ROS↓ in BMMs; TCA/OXPHOS proteins↓; RANKL-induced NF-κB, MAPKs, Akt signaling↓.	In vivo: Calvarial: BV/TV↑, OC number↓. OVX: Femoral BV/TV↑, Tb.N↑, Tb.Th↑, Tb.Sp↓. In vitro: OC marker↓; OC formation and bone resorption↓; Intracellular ATP↓.	[89]
Animal Model: Female Wistar rats, OVX-induced OP model (n = 9).	Intervention: Oral *Lepidium sativum* seed methanol extract. Dose & Route: 50 and 100 mg/kg, daily oral gavage for 12 wks. Safety alert: No overt toxicity.	Serum LDH and OCN↑; CTX-I and TRAP↓; MDA↓, SOD and GPX↑; RANKL/OPG ratio↓.	In vivo: Femur wet weight and compression strength↑; Cortical/trabecular thinning and marrow space widening↓; Histopathology score↓.	[90]
Animal Model: Female C57BL/6 mice, OVX-induced OP model (n = 10). Cellular Model: MC3T3-E1 pre-OB cell line.	Intervention: HIIT or lactate. Dose & Route: In vivo: HIIT (treadmill, 5 d/wk, 7 wks) or s.c. Nala (2 g/kg, 5 d/wk, 7 wks). In vitro: Nala (20 mM, 14 d). Safety alert: No overt toxicity.	Runx2, OSX, and OPN expression↑.	In vivo: BMD↑, BV/TV↑, Tb.N↑, Tb.Sp↓, Bone strength↑; OB number and bone formation↑. In vitro: ALP activity↑; Mineralization↑.	[91]
Animal Model: Male C57BL/6 mice, HFD model (n = 6). Cellular Model: BMMs, pre-OBs, OCs.	Intervention: Surgical (eWATx), pharmacological/local (clodronate liposomes, OPN-Ab), in vitro (rOPN, lactate). Dose & Route: In vivo: eWATx; local injections (clodronate/OPN-Ab) q3d, 8 wks. In vitro: rOPN (0.5 μg/mL); lactate. Safety alert: Well-tolerated.	OPN-integrin αvβ3 binding↑; MMP-9↑, Bone degradation↑; ATP6V0d2↓, Lactate/ATP in BMMs↑; Lipophagy↑; Osteoclastogenesis↓.	In vivo: HFD-induced bone loss↓; rBMAT accumulation↓. In vitro: rOPN: OC resorption↑. Lactate: Atp6v0d2 and osteoclastogenesis↓.	[92]
Human Observational: Osteomyelitis patients (n = 135). Animal Model: Male C57BL/6 mice, LPS-induced bone loss (n = 5). Cellular Model: MC3T3-E1 pre-OB cell line.	Intervention: Melatonin. Dose & Route: In vivo: i.p., Melatonin (5 mg/kg, 3×/wk, 1mo) + LPS. In vitro: Melatonin (100 nM) pre-treatment before LPS. Safety alert: No overt toxicity.	SIRT1↑; Mitophagy/mitochondrial dysfunction↓; HIF-1α stabilization↓; PDK1↓, p-PDH↓, Lactate production↓.	Human Observational: 43.9% hyperlactatemia; Gram-negative infection linked to blood lactate↑. In vivo: BV/TV↑, BMD↑, Tb.N↑, Tb.Th↑, Tb.Sp↓, OCN^+^ cells↑; Marrow IL-6, TNF-α, lactate↓. In vitro: Mineralization↑, ALP activity↑; osteogenic gene↑; IL-6 and TNF-α secretion↓.	[93]
Animal Model: Ducks, Cd-induced OP model (n = 6). Cellular Model: Duck primary BMSCs, OBs, and BMMs.	Intervention: Melatonin. Dose & Route: In vivo: CdCl_2_ (2 mg/kg diet) + Melatonin (0.2 mg/L in drinking water) for 5 mo. In vitro: Melatonin (0.1/1 μM) pre-treatment. Safety alert: No overt toxicity.	OB markers (OPN, OPG, COL1A1, Runx2) ↑; OC markers (RANKL, TRAP, NFATc1) ↓; Pyroptosis proteins (GSDMD, NLRP3, Casp-1) ↓; IL-18 and IL-1β↓.	In vivo: BV/TV↑, BMD↑, Tb.N↑, Tb.Th↑, Tb.Sp↓, SMI↓, TRAP^+^ OC number↓. In vitro: Rescued Cd-inhibited BMSC mineralization; Cd-induced OC formation and bone resorption↓; Cd-induced LDH and IL-1β release↓; OB death↓.	[94]
Animal Model: Female C57BL/6 mice, OVX-induced OP model. Cellular Model: Mouse BMMs and BMSCs.	Intervention: MCT1/2 inhibitor AZD3965, bone-targeting nanoparticle (PH/DPA@A). Dose & Route: In vivo: i.v., Free AZD3965 or nanoparticle (2 mg/kg, qod, 6 wks). In vitro: AZD3965 (0, 5, 10 μM). Safety alert: No overt toxicity.	OC markers (NFATc1, c-Fos, Integrin β3, CTSK, MMP-9) ↓; p-NF-κB and p-MAPK pathways↓; NFATc1 nuclear translocation↓; Extracellular lactate↓; Intracellular lactate↑; HK2, LDHA, PKM2↓.	In vivo: BV/TV↑, Tb.N↑, Tb.Th↑, Tb.Sp↓, N.Oc/BS, Oc.S/BS↓, Serum β-CTX and ACP5↓. In vitro: TRAP^+^ multinucleated cell and F-actin ring formation↓; Resorption pit area↓.	[95]
Animal Model: C57BL/6 mice (healthy/OVX); *Gpr81* KO mice (n = 6). Cellular Model: Mouse BMMs and BMSCs.	Intervention: Exercise (HIIT, LICT) and lactate. Dose & Route: In vivo: HIIT/LICT (treadmill, 5 d/wk, 8 wks); i.p., Nala (1 g/kg, 5×/wk, 8 wks) in OVX. In vitro: Nala (10 mM). Safety alert: No overt toxicity.	Bone surfaces *Gpr81*↑ after HIIT; Lactate (via *Gpr81*): p-TAK1 and p-p65↓ in BMMs; Wnt3, β-catenin, and OCN↑ in BMSCs.	In vivo (healthy): HIIT: BMD, microstructure, bone formation↑; Bone resorption↓ vs. LICT. In vivo (OVX): HIIT or lactate rescued bone loss (*Gpr81*-dependent). In vitro: Lactate: Osteoclastogenesis↓, Osteogenesis↑.	[96]
Human Observational: Female patients (n = 86). Animal Model: Female C57BL/6 mice, OVX model; Endothelial *Pkm2* KO mice (n = 4-5). Cellular Model: BMECs and BMSCs.	Intervention: AAV9-*Pkm2*, lactate, HIIT. Dose & Route: In vivo: s.c. Lactate (1.5 g/kg, q2d, 8 wks); i.v. AAV9-*Pkm2*; HIIT (treadmill, 5 d/wk, 8 wks). In vitro: Lactate (5 mM); *Mct1* siRNA; A485. Safety alert: No overt toxicity.	Endothelial PKM2 drives glycolysis and lactate production↑. Lactate: H3K18la↑ in BMSCs. H3K18la enriches at osteogenic gene promoters (*Col1a2*, *Comp*, *Enpp1*, *Tcf7l2*) to upregulate expression.	Human Observational: Serum lactate↓ in OP patients; H3K18la and osteogenic genes expression in patient BMSCs↓. In vivo: OVX and endothelial *Pkm2* KO: Bone loss↑; Vasculature↓, AAV9-*Pkm2*, lactate, or exercise: Bone mass and vessels↑. In vitro: Lactate or endothelial *Pkm2* OE rescued impaired OB differentiation caused by *Pkm2* KO BMECs.	[21]

**Abbreviations:** AAV9, adeno-associated virus serotype 9; ACP5, acid phosphatase 5, tartrate resistant; β-CTX, β-C-terminal telopeptide of type I collagen; BMMs, bone marrow-derived macrophages; BMECs, bone marrow endothelial cells; BV/TV, bone volume per tissue volume; Casp-1, caspase-1; CTSK, cathepsin K; Enpp1, ectonucleotide pyrophosphatase/phosphodiesterase 1; eWATx, epididymal white adipose tissue excision; GPX, glutathione peroxidase; i.p., intraperitoneal; i.v., intravenous; KD, knockdown; KO, knockout; MDA, malondialdehyde; Nala, N-acetyl-L-aspartic acid; NLRP3, NLR family pyrin domain containing 3; N.Oc/BS​, number of osteoclasts per bone surface; Oc.S/BS, osteoclast surface per bone surface; OE, overexpression; PH/DPA@A​, phenylboronic acid pinacol ester-Hyaluronic acid/DSPE-PEG-Asp8 @ AZD3965 nanoparticle; q2d, once every two days; qod, every other day; rBMAT, regulated bone marrow adipose tissue; s.c., subcutaneous; shRNA, short hairpin RNA; siRNA, small interfering RNA; SMI, structure model index; SOD, superoxide dismutase; Tb.N, trabecular number; Tb.Sp, trabecular separation; Tb.Th, trabecular thickness; Tcf7l2, transcription factor 7 like 2; ↑, increase or upregulation; ↓, decrease or downregulation.

**Table 2:** The potential of multiple strategies for targeting lactate and its upstream and downstream signaling in OA.

Model type and sample sizes	Treatments	Mechanism of action	Main outcomes	References
Human Observational: Paired cartilage from OA patients (n = 12). Animal Model: Male C57BL/6 mice, MLI-OA model; Chondrocyte-specific *Ldha* KO mice (n = 4-5). Cellular Model: Primary murine and human chondrocytes.	Intervention: Genetic (*Ldha* KO) and inhibitors (FX11, IKK2i). Dose & Route: In vitro: FX11 (40 μM); IL-1β (10 ng/mL). In vivo: i.p., IKK2i VIII (2 mg/kg, qod, 5 wks). Safety alert: No overt toxicity.	IL-1β induces NF-κB-dependent aerobic glycolysis: *Hk2*, *Ldha*↑; OXPHOS genes↓; Lactate↑, OCR↓. LDHA-NADH: ROS↑. IL-1β via IKK2/NF-κB: IκB-ζ↑; ROS stabilizes IκB-ζ protein. IκB-ζ essential for *Il6*, *Mmp13* expression.	Human Observational: OA cartilage: *Ldha*, *G6pd2*, *Mmp13*, *Nfkbiz*↑. In vivo: Chondrocyte-specific *Ldha* KO: OARSI score↓; MMP-13↓. Systemic IKK2i: OA progression↓. In vitro: IL-1β: *Il6*, *Mmp13*↑. LDHA inhibition (FX11, KO) or NF-κB inhibition: IL-1β-induced *Il6*, *Mmp13*↓; IκB-ζ protein↓; Intracellular ROS↓.	[72]
Human Observational: Plasma and cartilage from female OA patients (n = 10). Animal Model: Male C57BL/6 mice, HFD model (n = 5). Cellular Model: Primary mouse and human chondrocytes.	Intervention: Dietary (HFD), inhibitors (Oxamate, KG-548, TLR4i), siRNA. Dose & Route: In vivo: HFD; i.p. inhibitors [Oxamate (500 mg/kg/d), KG-548 (1 mg/kg/d), TLR4i (1 mg/kg/d), 14d]. In vitro: Stearic acid (200 μM), lactate (25 mM); inhibitors; siRNA. Safety alert: No mortality or adverse reactions.	Stearic acid: *Ldha* expression and lactate production↑. Lactate: HIF-1α stability and activity↑. HIF-1α: *Il6*, *Tnfα*, *Il1β*, *Vegf* mRNA↑. This axis is TLR4-independent.	Human Observational: Patient BMI correlates with plasma lactate↑; Cartilage HIF-1α protein↑; *Il6*, *Tnfα*, *Il1β*, *Vegf* mRNA↑. In vivo: HFD: Plasma cytokines, FFA, lactate↑; Cartilage *Il6*, *Tnfα*, *Il1β*, *Vegf* mRNA↑. Oxamate or KG-548: HFD-induced cytokine/VEGF expression↓. In vitro: Stearic acid/lactate: HIF-1α stability and activity↑. *Ldha* siRNA or Oxamate blocks stearic acid effects. *Hif1α* siRNA blocks cytokine/VEGF induction.	[75]
Human Observational: OA synovium. Animal Model: Male SD rats, ACLT + DMM OA model; IA lactate model (n = 5). Cellular Model: Primary human OA-FLS.	Intervention: IA lactate, siRNA-*Arg2*, inhibitors (Rapamycin, LY294002). Dose & Route: In vivo: IA lactate (200 mmol/L, 12 wks). In vitro: Lactate (20 mM); siRNA-*Arg2*; Rapamycin; LY294002. Safety alert: 20 mM lactate non-cytotoxic.	Lactate: *Arg2* expression↑. ARG2: mTOR/S6K1 pathway (p-mTOR, p-S6K1↑); p-eIF4B, p53, p21↑. p53/p21: SASP factors↑. Effect is ARG2 and mTOR-dependent.	Human Observational: *Arg2*↑ in OA synovium. In vivo: Surgical OA and IA lactate: synovitis score↑; MMP-3↑. IA lactate induces OA-like cartilage damage. *Arg2* expression↑ in synovium. In vitro: Lactate induces senescence (SA-β-gal^+^ cells↑, G1 arrest↑, ROS↑) and SASP factors↑. si-*Arg2* attenuates. Rapamycin/LY294002 inhibit lactate-induced mTOR pathway and SASP.	[97]
Human Observational: OA cartilage (n = 6). Animal Model: Male C57BL/6J mice, DMM-OA model; chondrocyte-specific *Mavs* KO mice; AAV5-*Mavs* OE (n = 5-6). Cellular Model: Primary mouse chondrocytes.	Intervention: Genetic (KO, AAV5-*Mavs*), IA L-lactate, inhibitors (BAY 11-7082, BX795). Dose & Route: In vivo: IA AAV5-*Mavs*; IA L-lactate (0.25 mg/kg). In vitro: Poly(I:C) (1.5 μg/mL), IL-1β (10 ng/mL), L-lactate (25 mM), BAY 11-7082, BX795. Safety alert: 1 mg/kg lactate induced bone damage; 0.25 mg/kg therapeutic.	Cytosolic mt-dsRNA: MAVS↑, NF-κB pathway (p-p65↑); *Mmp3/13* transcription↑; MMP-3/13 protein↑; ACAN and COL2↓. L-lactate: MAVS↓, NF-κB and MMP-3/13↓.	Human Observational: MAVS aggregation and cytosolic dsRNA in OA chondrocytes. In vivo: *Mavs* deficiency: OARSI score↓, synovitis↓, BV/TV↑, Tb.Th↑, Osteophyte formation↓. *Mavs* OE exacerbates. L-lactate: Cartilage degradation and synovitis↓. In vitro: Poly(I:C)/IL-1β: MAVS aggregation; NF-κB, *Mmp3*/*13*↑ in WT. BAY 11-7082 blocks induction. L-lactate inhibits MAVS aggregation and IL-1β-induced MMP-3/13.	[98]
Human Observational: TMJOA synovium/synovial fluid (n = 20 patients, 8/20 controls). Cellular Model: Primary human TMJOA SFs.	Intervention: LDHA inhibitor (GSK2837808A). Dose & Route: In vitro: GSK2837808A (0-20 μM). Safety alert: GSK≤20 μM no cytotoxicity.	GSK: Active LDHA↓; Glucose uptake↓; Intracellular lactate↓; ATP/AMP ratio↑; p-AMPK↓, HAS2 protein↑; IL-1β secretion↓.	Human Observational: TMJOA: synovial LDHA protein↑; IL-1β↑, HAS2↓. Synovial fluid: active LDHA↑, Lactate↑, IL-1β↑, HA↓. In vitro: GSK: Lactate secretion↓; IL-1β↓, HA concentration↑.	[99]
Animal Model: Male Wistar rats, ACLT-OA model (Sham n = 11, ACLT n = 10, Tx n = 8).	Intervention: IA oxamate (LDHA inhibitor). Dose & Route: IA oxamate (0.25 or 2.5 mg/kg) qwk, 5 wks, starting 10 wks post-surgery. Safety alert: No overt toxicity.	Oxamate: Glycolytic gene (*Glut1*, *Glut3*, *Hk2*, *Pkm2*, *Pdk1*, *Pdk2*, *Ldha*↓) in cartilage. Oxamate: TUNEL^+^ apoptotic cells↓.	In vivo: Oxamate: Weight-bearing deficit↓; Joint swelling↓; OARSI score↓; Cartilage structure and ECM↑; TUNEL^+^ chondrocytes↓.	[100]
Animal Model: Male C57BL/6J mice, DMM-OA model; chondrocyte-specific *Glut1* KO mice (n = 8). Cellular Model: ATDC5 cell line.	Intervention: Pharmacological (5-HMF, Celebrex), genetic (*Glut1* KO), and cytokine (IL-1β). Dose & Route: In vivo: i.p. 5-HMF (7.5-30 mg/kg/d) or oral Celebrex (26 mg/kg/d), 8 wks; tamoxifen-induced *Glut1* KO. In vitro: IL-1β (10 ng/mL), 5-HMF, siRNA-*Glut1*. Safety alert: 5-HMF≤200 μg/mL non-toxic.	5-HMF: Glut1/HK1/LDHA axis↓; *Col2a1*↑, *Mmp13*↓. Effects abolished in *Glut1*-KO chondrocytes.	In vivo: 5-HMF: OARSI score↓; BV/TV↓, Tb.Th↓. Cartilage: *Col2a1*↑, MMP-13↓, Glut1, HK1, LDHA protein↑. Absent in *Glut1* KO. In vitro: 5-HMF reverses IL-1β effects (*Col2a1*↓, *Mmp13*↑, *Ldha*↓). siRNA-*Glut1* abolishes rescue.	[101]
Animal Model: Male New Zealand rabbits, immobilization-induced KOA (n = 6).	Intervention: EA. Dose & Route: EA at knee acupoints (2/100 Hz, 2 mA, 20 min/session, qod, 3 wks). Safety alert: Standard EA.	EA: Glycolytic enzymes (*Glut1*, *Pkm2*, *Ldha*↓) in cartilage; cartilage lactate content↓.	In vivo: EA: Lequesne MG score↓; Mankin score↓; Cartilage thickness↑. Synovial microcirculation (perfusion↑, velocity↑) and synovial fluid oxygen↑. Cartilage lactate↓.	[102]
Human Observational: Human cartilage. Animal Model: Male SD rats, IA Dex model (n = 8). Cellular Model: Primary HAC from OA (n = 3).	Intervention: IA Dex, antioxidants (NAC, α-KG), *Mct4* OE, lactate. Dose & Route: In vivo: IA Dex (0.5 mg/kg), 2×/wk, 8 wks. In vitro: Dex (250-1000 nM), lactate (0-500 mM), NAC (5 mM), α-KG (2 mM), *Mct4* OE plasmid. Safety alert: Dex≥1500 nM/48h or≥500 nM/72h toxic. Lactate>25 mM toxic.	Dex: mtDNA↓, OXPHOS genes↓; ΔΨm↓, ROS↑, H3K4me3 at *Mct4* promoter↓; *Mct4* mRNA and protein↓; Intracellular lactate↑; Extracellular lactate↓. Antioxidants or *Mct4* OE reverse.	Human Observational: IA Dex: *Mct4* in rat cartilage↓. In vivo: IA Dex: Cartilage GAG and anabolic gene (*Col2a1*, *Acan*↓). In vitro: Dex: COL2A1, ACAN↓; MMP13↑, Senescence (SA-β-gal↑) and ECM loss. *Mct4* OE reverses matrix↓, ROS↑, Lactate accumulation. NAC and α-KG alleviate Dex effects.	[103]
Human Observational: Serum and synovial fluid from OA (n = 40) and controls (n = 22); OA cartilage. Animal Model: Male SD rats, IA lactate; ACLT + DMM; immunodeficient model (n = 5). Cellular Model: Primary HAC from OA.	Intervention: Lactate, inhibitors (GLX351322, LY294002, MK2206, 2-DG, NAC), siRNA (si-*Gpr81*). Dose & Route: In vitro: Lactate (20 mM), GLX351322, LY294002, MK2206, 2-DG, NAC (5 mM), si-*Gpr81*. In vivo: IA lactate (200 mmol/L) qwk, 4/12 wks; ACLT + DMM; co-injection GLX351322. Safety alert: 20 mM lactate optimal non-toxic dose.	Lactate↑ in OA. Lactate: GPR81, MCT1-4, SLC5A8/12↑, PPP↑, NADPH↑. Lactate (via GPR81): PI3K/Akt (p-PI3K ↑, p-Akt ↑), NOX4↑, Intracellular ROS↑; Chondrocyte damage↑.	Human Observational: Serum and synovial fluid lactate↑ in OA. In vivo: IA lactate: OA-like damage (Mankin and OARSI scores↑). Worsens surgical OA. GLX351322 attenuates. In vitro: Lactate: Catabolic genes (*Mmp3/13*, *Adamts-4*↑); Inflammation↑, Hypertrophy markers↑; Senescence↑. GLX351322, NAC, or si-*Gpr81* reverse.	[23]
Human Observational: Synovial tissues/fluid from OA patients and controls (n = 30/188). Animal Model: Male C57BL/6 mice, DMM-OA; AAV-sh*Fstl1*; recombinant FSTL1 (n = 6). Cellular Model: ATDC5 cells; primary HAC.	Intervention: Recombinant FSTL1, AAV-sh*Fstl1*, oxamate, lactate, inhibitors (Paxalisib). Dose & Route: In vivo: IA FSTL1 or oxamate (50 mmol/L); IA AAV-sh*Fstl1*. In vitro: FSTL1 (40 ng/mL), lactate (10 mM), Paxalisib (70 nM), oxamate (50 mmol/L). Safety alert: Paxalisib≤125 nM and lactate≤12.5 mmol/L non-toxic.	FSTL1: PI3K/AKT/mTOR↑; HIF-1α↑, Glycolysis (*Ldha*, *Hk2*, *Pkm*↑); Lactate accumulation↑. Lactate: H3K18la↑, Fibrosis genes (*Itga6*, *Cxcl10*, *Parp16*) transcription↑. Paxalisib or oxamate: H3K18la and fibrosis↓.	Human Observational: Synovial fluid FSTL1↑ in OA, correlated with VAS, KL grade, age, BMI. In vivo: FSTL1 exacerbates DMM-induced OA (OARSI score↑; osteophyte↑, Cartilage degradation↑; COL2/SOX9; COL1/MMP-13↑). AAV-sh*Fstl1* attenuates. Oxamate alleviates fibrosis. In vitro: FSTL1: Fibrosis, glycolysis (ECAR↑, OCR↓, lactate↑). Paxalisib or oxamate blocks. Lactate mimics. H3K18la↑ at fibrosis gene promoters.	[104]
Animal Model: Male SD rats, ACLT-OA; IA lactate co-injection (n = 8). Cellular Model: Primary rat chondrocytes.	Intervention: Songorine, CXB, lactate, IL-1β, and *Pfkfb3* OE. Dose & Route: In vivo: IA Songorine (10-50 μM) or CXB (2 mg/mL) qwk, 4 wks; IA lactate (20 mM) co-injection. In vitro: IL-1β (10 ng/mL), Songorine (50 μM), lactate (20 mM), *Pfkfb3* OE. Safety alert: No systemic toxicity.	Songorine: PFKFB3, HK2, LDHA↓; Glycolytic flux (ECAR↓, 13C-lactate↓); Intracellular lactate↓; H4K12la and its enrichment at promoters of *Il6*, *Mmp9*, *Pfkfb3*↓; TNF-α, NF-κB, IL-17, IL-6, MMP-9↓.	In vivo: Songorine: OARSI score↓; BMD↓, BV/TV↓, superior to CXB. PFKFB3, LDHA, IL-6↓; SOX9, COL2A1↑. Lactate abolishes protection. In vitro: Songorine: Lactate↓, TCA flux↑; IL-6, MMP-9↓; Anabolic markers↑. Lactate or *Pfkfb3* OE reverses Songorine.	[105]
Animal Model: Male C57BL/6 mice, DMM-OA (n = 8). Cellular Model: Primary mouse chondrocytes.	Intervention: siRNA-*Ldhb*, Nala, Oxamate, *Acsl4* OE. Dose & Route: In vivo: IA si-*Ldhb* (20 μmol), 2×/wk, 8 wks; IA Nala (10 mg/kg) co-injection. In vitro: IL-1β (10 ng/mL), si-*Ldhb*, Nala (5 mM), Oxamate (5 mM), *Acsl4* OE. Safety alert: No overt IA toxicity.	LDHB and H3K18la↑ in OA. LDHB depletion or Oxamate: H3K18la enrichment at *Acsl4* promoter↓. H3K18la: *Acsl4* promoter↑. *Ldhb* depletion or inhibition: ACSL4 protein↓.	In vivo: si-*Ldhb*: serum CTX-II, COMP↓, Cartilage matrix degradation↓. Nala reverses. si-*Ldhb* partially restores cartilage matrix proteins. OA-induced iron, Fe^2+^, lipid ROS↓. In vitro: IL-1β: Viability, COL2/ACAN↓; Total iron, Fe^2+^, lipid ROS↑. si-*Ldhb* reverses; Nala abolishes.	[106]
Animal Model: Male SD rats, ACLR model (n = 12). Cellular Model: Rat BMSCs and CSPCs.	Intervention: BMSC-Exos ± *Ldha* KD, Nala, FX11, genetic manipulation. Dose & Route: In vivo: IA BMSC-Exos or BMSC-Exos-sh-*Ldha* post-ACLR. In vitro: sh-*Ldha*/sh-*Bmp7*; Nala (5-20 mM); FX11 (10 mM); Smad1/5/8 DN plasmid. Safety alert: Standard procedures.	BMSC-Exos deliver LDHA: Lactate in CSPCs↑. H3K18la enrichment at *Bmp7* promoter: BMP7 expression↑; p-Smad1/5/8 ↑. Effects are LDHA and lactate-dependent.	In vivo: BMSC-Exos promote cartilage repair; BMSC-Exos-*shLdha* attenuates. In vitro: BMSC-Exos: lactate, H3K18la, BMP7, p-Smad1/5/8↑. *shLdha* in BMSC-Exos or CSPCs abolishes. Lactate mimics, FX11 inhibits. Smad1/5/8 DN blocks BMP7 signaling.	[107]
Human Observational: OA and control cartilage. Animal Model: Male C57BL/6 mice, DMM; non-surgical IA lactate model. Cellular Model: Primary mouse chondrocytes; ATDC5.	Intervention: Lactate, DCA, A485, IL-1β, genetic manipulation of *Ugdh*. Dose & Route: In vivo: IA lactate (25 mM) in non-surgical mice; IA DCA (20 mM) or A485 (20 mM) post-DMM. In vitro: Lactate (25 mM), IL-1β (10 ng/mL), DCA (20 mM), A485 (5-20 μM), siRNA- *Ugdh*, *Ugdh* mutants. Safety alert: Not specified.	Lactate induces pan-lactylation, lactylates UGDH at K6. K6la: UGDH activity↓; UDP-Glc to UDP-GlcA conversion and GAG synthesis↓. UGDH nuclear-to-cytoplasmic translocation↑ via enhanced CRM1 and weakened KPNA2 binding. UGDH-STAT1 interaction↓; STAT1-mediated MAP3K8 transcription↑ and p38 MAPK pathway activation.	Human Observational: UGDH lactylation↑ in OA cartilage. In vivo: IA lactate: OA-like destruction, proteoglycan loss, OARSI score↑. DCA or A485 treatment alleviates DMM-induced OA progression. In vitro: Lactate/IL-1β: UGDH lactylation↑, GAG synthesis↓, ECM catabolism↑, apoptosis↑. UGDH K6R OE (vs. WT) more effectively rescues these phenotypes. A485 inhibits UGDH lactylation and reverses catabolic/pro-apoptotic effects.	[108]
Animal Model: Adult C57BL/6 mice, collagenase-induced OA; WT and *Ldha* KO (CKO) (Total n = 24). Cellular Model: Primary mouse articular chondrocytes.	Intervention: Genetic KO of *Ldha*, LPS, and pharmacological (lipoic acid, 2-DG). Dose & Route: In vivo: Collagenase IA injection. In vitro: LPS (5 μg/mL, 24h); lipoic acid (LA, 5 mM, 1h); 2-DG (10 mM, 1h); siRNA-*Ldha*. Safety alert: Standard.	*Ldha* and pan-lysine/H3K18la↑ in OA. *Ldha*-mediated H3K18la enrichment at *Tpi1* promoter. TPI1 K69 mutation ameliorates LPS-induced glycolysis.	In vivo: *Ldha* KO: OARSI score↓. Partially restores proteoglycan, cartilage thickness, chondrocyte number. OA-induced *Col2*↓, *Mmp13*↑. In vitro: *Ldha* KD reverses LPS-induced *Col2*↓ and *Mmp1*3 mRNA↑. LPS-induced glucose consumption, lactate, ECAR↓. TPI1 K69 mutation ameliorates LPS-induced changes in *Col2* and *Mmp13*.	[109]

**Abbreviations:** 2-DG, 2-deoxy-D-glucose; ACLT, anterior cruciate ligament transection; AMPK, adenosine 5′-monophosphate-activated protein kinase; CRM1, chromosome region maintenance 1; CSPCs, chondrogenic stem/progenitor cell; Cxcl10, C-X-C​ motif chemokine ligand 10; CXB, celecoxib; ΔΨm, mitochondrial membrane potential; DHI%, disc height index percentage; DMM, destabilization of the medial meniscus; ECAR, extracellular acidification rate; FFA, free fatty acid; HA, hyaluronic acid; GAG, glycosaminoglycan; HAC, human articular chondrocytes; HAS2, hyaluronan synthase 2; HFD, high-fat diet; IA, intra-articular; IKK2, IκB kinase 2; IκB-ζ, inhibitor of κB ζ; Itga6, integrin alpha-6; KEAP1, Kelch-like ECH-associated protein 1; KOA, knee osteoarthritis; KPNA2,karyopherin alpha 2​; Lequesne MG score, lequesne algofunctional index; Mankin score, Mankin histological-histochemical grading system; MLI-OA, meniscal-ligamentous injury-induced osteoarthritis; mt-dsRNA, mitochondrial double-stranded RNA; NAC, N-Acetyl-L-cysteine; NRF2, nuclear factor erythroid 2-related factor 2; OARSI, Osteoarthritis Research Society International; OCR, oxygen consumption rate; Parp16, Poly(ADP-ribose) polymerase family member 16; PPP, pentose phosphate pathway; qwk, once weekly; SA-β-gal, senescence-associated β-galactosidase; SASF, synovial fluid; SF, synovial fibroblasts; SLC5A8/12, solute carrier family 5, member 8/12; TLR4, toll-like receptor 4; TMJOA, temporomandibular joint osteoarthritis; TPI1, triosephosphate isomerase 1; TUNEL, terminal deoxynucleotidyl transferase dUTP nick end labeling; UDP-Glc, uridine diphosphate glucose; UDP-GlcA, uridine diphosphate glucuronic acid.

**Table 3:** The potential of multiple strategies for targeting lactate and its upstream and downstream signaling in RA.

Model type and sample sizes	Treatments	Mechanism of action	Main outcomes	References
Human Observational: Synovial fluid from 27 RA to 22 HC; Synovial tissues from 5 RA to 6 OA. Animal Model: WT and *Cd31* KO mice, CIA. Cellular Model: Primary mouse synovial microvascular ECs.	Intervention: L-lactate, *Cd31* KO, ITIM mutants, CD31 Ab, 2-DG. Dose & Route: In vitro: Lactate (100 nM, 45 min-6h); 2-DG. In vivo: CD31 Ab in CIA. Safety alert: Standard, no overt toxicity.	Lactate triggers CD31 activation, SHP2 recruitment, glycolytic shift (glucose uptake↑, *Glut1*/*Hk2*↑, ECAR↑). OXPHOS suppression: OCR↓. CD31/Lactate: STAT3 translocation↑; p-AMPK↑, p-mTOR↓; Autophagy markers↑. ITIM mutant reverse.	Human Observational: Synovial fluid lactate↑ vs. HC; correlates with progression. CD31^+^ vessels correlate with activity. Autophagy and HK2↑ vs. OA. In vivo: *Cd31* KO/ITIM mutant mice: Disease severity↓; ATG7↓, NADH dehydrogenase↑. In vitro: Lactate in WT ECs: Glycolysis↑, STAT3 translocation↑; Autophagy↑. Lactate in *Cd31* KO ECs: Oxidative metabolism↑. ITIM mutant reconstitution reverses.	[110]
Animal Model: Male SD rats, AIA (n = 8). Cellular Model: RAW264.7 cells, primary mouse BMDMs.	Intervention: SNEDDS-TG, TG, MTX, siRNA. Dose & Route: In vivo: Oral SNEDDS-TG (100 mg/kg/d, 30d). In vitro: TG (50-200 μM); 2-DG; siRNA-*Ldha*. Safety alert: TG ≤ 200 μM no cytotoxicity. No organ alterations.	TG blocks M1 metabolic switch: ECAR↓, Lactate↓, OCR↑. TG: *Ldha*, *Pdh*, *Hif-1α*, *Glut1*↓. *Ldha* siRNA abolishes TG effects. 2-DG attenuates.	In vivo: SNEDDS-TG: AI↓; Paw volume↓. Inflammation and bone destruction↓; Serum TNF-α and IL-1β↓; CD11b^+^ CD86^+^ M1↓. In vitro: TG: M1 polarization and cytokine mRNA↓.	[111]
Animal Model: Male DBA/1 J mice, CIA (n = 5). Cellular Model: Human FLS.	Intervention: HAP-1NPDC + LND, DC, LND. Dose & Route: In vivo: i.v. NPs q3d, 5 doses. In vitro: NPs. Safety alert: No cytotoxicity. No organ alterations.	DC inhibits GLUT-1; LND inhibits HK-2. Synergy suppresses FLS metabolism; lactate↓. NPs inhibit NF-κB in co-cultured macrophages: p-IκBα↓, p65↓.	In vivo: NPs: joint accumulation↑; Ankle thickness↓; Arthritis score↓; Bone erosion↓; Synovial cartilage damage↓. Synovial GLUT-1 and HK-2↓; FAP-α^+^ FLS↓; CD86^+^ M1↓. In vitro: NPs: FLS viability↓; Glucose uptake↓; Lactate↓, ATP↓, FLS invasion and migration↓; MMP-2↓. Co-culture: M1 polarization and TNF-α and IL-1β↓.	[112]
Human Observational: Synovial tissue from 20 RA to 20 OA. Animal Model: SD rats, CIA (n = 6). Cellular Model: Human FLS.	Intervention: Shikonin, TNF-α, SC79, sh-*Pkm2*. Dose & Route: In vivo: i.p. Shikonin (5 mg/kg, q3d, 30d). In vitro: TNF-α (10 ng/mL, 24h); Shikonin; SC79; sh-*Pkm2*. Safety alert: Shikonin is PKM2 inhibitor. No overt toxicity.	PKM2↑ in RA vs. OA. PKM2 promotes FLS glycolysis: Glucose uptake↑; ATP↑, Lactate↑, ECAR↑; cytokine (TNF-α, IL-1β, IL-6) release↑; p-Akt/p-mTOR↑. *Pkm2* KD reverses. SC79 rescues.	Human Observational: PKM2↑ in RA synovium vs. OA. In vivo: Shikonin: AI↓, Paw swelling↓; Synovial and cartilage damage↓; Synovial PKM2, p-Akt, p-mTOR↓; TNF-α, IL-1β, IL-6↓; Bone erosion↓, BV/TV, BMD, Tb.Th↑. In vitro: TNF-α: PKM2↑, Glycolysis↑. *Pkm2* KD: TNF-α-induced glycolysis↓; cytokines↓, p-Akt/p-mTOR↓. SC79 reverses.	[113]
Animal Model: Male Lewis rats, AIA (n = 6). Cellular Model: RAW 264.7 cells.	Intervention: Tan IIA, MTX, GF, RANKL. Dose & Route: In vivo: Oral Tan IIA (7.5-30 mg/kg/d) or MTX (0.2 mg/kg, 2×/wk) for 30d. In vitro: RANKL (50 ng/mL); Tan IIA (10-40 μM); GF (20 μM). Safety alert: No overt toxicity.	Tan IIA binds LDHC, inhibits LDHC activity. Tan IIA: NAD^+^/NADH ratio↓; Intracellular ROS in OCs↓. GF phenocopies Tan IIA effects.	In vivo: Tan IIA (30 mg/kg): Paw swelling↓; Arthritis score↓; Pain threshold↑; Synovitis and histopathology score↓; BV/TV↑, Tb.N↑, Tb.Th↑; Serum MMP-9, TRAP, CTSK, IL-17↓. In vitro: Tan IIA: RANKL-induced TRAP^+^ OCs ↓; F-actin rings↓. Tan IIA^+^ GF: OC markers↓.	[114]
Human Observational: Synovial tissue from 12 RA to 9 OA. Animal Model: Mice, CIA (n = 8). Cellular Model: Primary human RA and OA FLS.	Intervention: PFK15, TNF-α, Si- *Pfkfb3*, L-lactic acid. Dose & Route: In vitro: PFK15 (0.5-5.0 μM, 4h); TNF-α (10 ng/mL); Si-*Pfkfb3* (100 nM); lactate (10 mM). In vivo: i.p. PFK15 (25 mg/kg, qod, 14d). Safety alert: PFK15 ≤ 10 μM non-cytotoxic. No in vivo toxicity.	PFKFB3↑ in RA vs. OA. PFK15/Si-*Pfkfb3*: Glucose uptake↓; Lactate↓, F2,6BP↓. PFK15: TNF-α-induced p65 translocation↓; p-IKKβ/p-IκBα↓; p-p38, p-JNK, p-ERK↓. Lactate reverses.	Human Observational: PFKFB3 protein↑ in RA synovium vs. OA. In vivo: PFK15: Arthritis score↓; Ankle swelling↓; Synovitis and bone damage↓; Serum and synovial IL-6↓. In vitro: PFK15/Si-*Pfkfb3*: TNF-α-induced *Il6*, *Il8*, *Ccl2*, *Cxcl1*0 mRNA↓; FLS migration, invasion, proliferation↓. Lactate reverses.	[115]
Human Observational: Synovial tissue from 12 RA to 12 OA. Animal Model: Male DBA/1 mice, CIA (n = 8). Cellular Model: Primary human RA FLS.	Intervention: NH125, TNF-α, IL-1β, lactate, *Eef2k* siRNA. Dose & Route: In vitro: NH125 (0.1-1.0 μM, 24h); TNF-α/IL-1β (10 ng/mL); *Eef2k* siRNA (100 nM). In vivo: Daily i.p. NH125 (1 mg/kg/d, 14d). Safety alert: NH125 ≤ 5.0 μM non-cytotoxic. No serum alterations.	eEF2K↑ in RA vs. OA. TNF-α and IL-1β: eEF2K↑. NH125 and *Eef2k* siRNA: Glucose uptake↓; F2,6BP↓, lactate↓; TNF-α-induced p-IKK, p-IκBα and p65 translocation↓; p-Akt↓. Lactate reverses.	Human Observational: eEF2K protein↑ in RA synovium vs. OA. In vivo: NH125: Arthritis score↓; Paw thickness↓; Synovitis and bone erosion↓; Serum cytokines↓. In vitro: NH125 and eEF2K siRNA: TNF-α-induced cytokines↓; FLS proliferation, migration, invasion↓.	[116]
Animal Model: DBA/1 mice, CIA (n = 8). Cellular Model: RAW264.7 cells, chondrocytes, FLS.	Intervention: Novel delivery and Photocatalytic: HTON + NIR laser. Dose & Route: In vivo: IA HTON + NIR laser (0.3 W/cm^2^) qwk, 5 sessions. Safety alert: HTON high viability. No organ alterations. NIR safe.	Photocatalysis: H_2_ generation + lactate depletion. H_2_ scavenges ROS: M1 polarization (iNOS↓), M2 phenotype (CD206↑). Lactate depletion inhibits inflammatory phenotypes. Disrupts M1-FLS loop.	In vivo: HTON + NIR: Paw swelling↓; Arthritis score↓; Bone erosion↓; Synovitis↓, Cartilage preserved. Synovial lactate↓; H_2_↑. Synovial proinflammatory markers↓; Macrophage shift (M1↓, M2↑). In vitro: Lactate induces proinflammatory phenotypes. HTON treatment reverses, inhibits MMP3.	[117]
Human Observational: RA synovial tissue and fluid (IF n = 8, tissue n = 20; PEAC n = 36). Cellular Model: Primary RA fibroblasts, macrophages.	Intervention: Nala, phloretin, TNF-α, conditioned medium. Dose & Route: In vitro: Lactate (10 mM, 24h); phloretin (41 μM); TNF-α (10 ng/mL); conditioned medium (10%). Safety alert: No increased cell death.	*Mct1*/*4*↑ in RA. Macrophages: *Mct4* high; Fibroblasts: *Mct1*/*4*. Fibroblasts + lactate: Glycolysis↑, Basal respiration↑; Spare capacity↓. Macrophages + lactate: Glycolysis↓. Lactate enhances TNF-α in fibroblasts, inhibits in macrophages.	Human Observational: *Mct4* mRNA correlates with synovitis score and DAS28. In vitro: Lactate: Fibroblast migration↑; Macrophage chemotaxis↓. With TNF-α and lactate: Fibroblast IL-6↑; Macrophage IL-6↓.	[118]
Human Observational: Synovial tissue and fluid from 16 RA to 9 OA. Animal Model: Male DBA/1 mice, CIA. Cellular Model: Primary human RASFs and OASFs.	Intervention: *Mct4* siRNA, control siRNA. Dose & Route: In vitro: Transfection. In vivo: IA *Mct4* siRNA complexed, 2×/wk, 4 wks. Safety alert: No overt toxicity. *Mct4* KD induces apoptosis in RASFs.	*Mct4*↑ in RASFs vs. OASFs. *Mct4* KD: Lactate export↓; Intracellular lactate↑; pH↓.	Human Observational: RA SF: pH↓, lactate↑ vs. OA; pH correlates inversely with DAS28 and lactate. In vivo: *Mct4* siRNA: AI↓; Joint swelling↓; Synovitis↓, Apoptotic cells in synovium↑. In vitro: *Mct4* KD: RASFs proliferation↓; IL-6 and MMP-3↓; Invasion↓.	[119]
Human Observational: PBMCs and SFMCs from RA patients; inflamed tonsils. Animal Model: DBA/1 mice, G6PI-induced arthritis (n = 6). Cellular Model: Primary human CD4^+^ T cells.	Intervention: SLC5A12 blocking Ab, lactate, pathway inhibitors, *Slc5a12* KO mice. Dose & Route: In vivo: i.p. SLC5A12 Ab (100 μg) on days 0, 3, 7, 10, 14. In vitro: Lactate (10 mM); inhibitors. Safety alert: No overt toxicity reported.	Lactate/inflammation upregulate *Slc5a12*. *Slc5a12*-mediated lactate uptake: Glycolysis (glucose uptake↓, ECAR↓); Mitochondrial oxidation↑; Metabolism to PPP and FAS. Lactate: PKM2 nuclear translocation↑; p-STAT3↑. FAS enhances p-STAT3.	Human Observational: SLC5A12 on CD4^+^ T cells in RA SF; correlates with synovial T cell score. In vivo: SLC5A12 Ab: Arthritis score↓; Paw swelling↓; Synovitis↓. In vitro: Lactate: IL-17A↑, RORγt↑; CD4^+^ T cell chemotaxis↓. Inhibition blocks lactate effects. *Slc5a12* KO cells resist lactate.	[120]
Human Observational: PBMCs from 68 RA patients and 34 HC; synovial tissue and fluid. Animal Model: Male Wistar rats, CIA. Cellular Models: RA FLS, THP-1 cells.	Intervention: DCA, rotenone, TNF-α, lactate, si-*Sirt3*, HKL. Dose & Route: In vivo: Oral DCA (200 mg/kg/d) or rotenone (10 mg/kg/d) for 21d. In vitro: TNF-α; lactate (10 mM); DCA; si-*Sirt3*; HKL. Safety alert: Rotenone side effects. DCA well-tolerated.	Lactate and H3K18la↑ in RA. TNF-α and lactate: Intracellular lactate↑; H3K18la↑. H3K18la regulates chemokine genes. SIRT3↓ in RA. *Sirt3* KD: H3K18la↑; HKL: H3K18la↓. SIRT3 KD in macrophages activates FLS.	Human Observational: SF lactate↑; H3K18la↑ in RA synovium; SIRT3↓ in PBMCs, correlates with DAS28. In vivo: DCA: Arthritis score↓; Paw swelling↓; Joint damage↓; Synovial H3K18la↓. In vitro: Lactate + TNF-α: RA FLS activation↑; DCA inhibits. *Sirt3* KD: H3K18la↑, FLS activation↑. SIRT3 activation inhibits.	[121]
Human Observational: RA synovial tissue. Animal Model: Male SD rats, AIA (n = 5). Cellular Models: Primary RA FLS, THP-1.	Intervention: GAS, OXA, 2-DG, *Kat8* OE, si-*Kat8*, LPS. Dose & Route: In vivo: Oral GAS (2/20 mg/kg/d) or DEX (5 mg/kg/d) for 50d. In vitro: GAS (5-20 μM); LPS; OXA; 2-DG. Safety alert: GAS≤20 μM non-cytotoxic. No oral toxicity.	GAS: glycolysis (ECAR↓, lactate↓); pan-lysine and H3K9la↓. GAS binds/destabilizes KAT8. *Kat8* OE: H3K9la↑, IL-6↑. si-*Kat8*: H3K9la↓, IL-6↓.	In vivo: GAS (20 mg/kg): Joint swelling↓; Arthritis score↓; Synovitis↓, Synovial H3K9la and IL-6↓. In vitro: GAS: LPS-induced IL-6; MMP-1/13↓. FLS lactate correlates with IL-6 and MMP-13. OXA/2-DG mimic GAS.	[122]
Animal Model: SD rats, CIA. Cellular Model: Primary human RA-FLS and rat CIA-FLS.	Intervention: ART, si-*Pkm2*, pPKM2, si-*p300*. Dose & Route: In vitro: ART (0-6.0 μM, 16-24h). In vivo: Oral ART (2.5-10 mg/kg, q2d, 36d) or MTX (1 mg/kg). Safety alert: ART≤6 μM no cytotoxicity. No oral toxicity.	ART binds PKM2: Enhances p300-mediated PKM2 lactylation↑. ART alters conformation, inhibits nuclear translocation. ART induces S-phase arrest (Cyclin B1↓). Effects p300-dependent.	In vivo: ART (5, 10 mg/kg): AI↓; Swelling↓, Gait↑, Bone destruction↓; Synovitis↓, FLS markers and TNF-α↓; TRAP^+^ OCs↓. In vitro: ART: RA-FLS/CIA-FLS viability, migration, DNA replication↓; *Pkm2* OE reverses. *p300* KD reverses ART effects.	[123]
Human Observational: RA, HC, GA, PsA FLS, synovium, serum. Animal Models: Male DBA/1 mice (CIA, n = 10); SCID mice; *Nfatc2* KO mice (CAIA, n = 5). Cellular Model: Primary human RA-FLS.	Intervention: FX-11, AZD-3965, lactate, siRNA/shRNA, lentivirus-sh*Nfatc2*, FLS-specific *Nfatc2* KO. Dose & Route: In vitro: Lactate (10 mM); FX-11 (10 μM); AZD-3965 (100 nM). In vivo: i.p. FX-11 (2 mg/kg) or AZD-3965 (100 mg/kg) daily, 28d; IA LV-sh *Nfatc2*; tamoxifen-induced KO. Safety alert: No overt toxicity.	H3K9la↑ in RA. H3K9la enriches at *Nfatc2* promoter. *Ldha* KD/inhibition: Lactate↓, H3K9la↓, NFATc2↓. MCT1 inhibition blocks lactate-induced H3K9la and NFATc2↑. NFATc2 promotes IL-6, IL-8, MMP-9/13. Anti-H3K9la autoantibodies correlate with DAS28.	Human Observational: H3K9la and NFATc2↑ in RA synovium and FLS. Serum anti-H3K9la autoantibodies↑ in RA, correlate with DAS28. In vivo: FX-11/AZD-3965: Disease severity↓; Inflammation↓, Serum cytokines↓. *Nfatc2* KD/KO: Disease severity↓. *Nfatc2* KD in FLS↓ erosive capacity in SCID mice. In vitro: Lactate/H3K9la via NFATc2 promotes FLS migration, invasion, cytokines, MMPs. *Ldha*/*Mct1* inhibition or *Nfatc2* KD blocks effects.	[124]

**Abbreviations:** AI, arthritis index; ATG7, autophagy-related gene 7; BMDMs, bone marrow-derived macrophages; CAIA, collagen antibody-induced arthritis; DAS28, Disease Activity Score in 28 joints; DC, diclofenac; DCA, dichloroacetate; HKL, Honokiol; ERK, extracellular signal-regulated kinase​; F2,6BP, fructose-2,6-bisphosphate; FAP-α, fibroblast activation protein-alpha; GA, gouty arthritis; GAS, Gastrodin; GF, Galloflavin; H_2_, Hydrogen (molecule); HAP-1NPDC + LND, HAP-1 peptide-modified dual polymer-prodrug conjugates-assembled nanoparticles system co-loaded with DC and LND; HC, healthy controls; HTON + NIR, hydrogen-doped titanium dioxide nanorods + near-infrared (laser irradiation); IκBα, inhibitor of kappa B alpha; IKKβ, inhibitor of nuclear factor kappa-B kinase subunit beta; iNOS, inducible nitric oxide synthase; JNK, c-Jun N-terminal kinase; LND, Lonidamine; MTX, methotrexate; NPs, nanoparticles; OASFs, osteoarthritis synovial fibroblasts; OXA, Oxamate; PBMCs, peripheral blood mononuclear cells; PFK15, 3-(3-Pyridinyl)-1-(4-pyridinyl)-2-propen-1-one; PsA, psoriatic arthritis; q3d, once every three days; RASFs, rheumatoid arthritis synovial fibroblasts; RORγt, RAR-related orphan receptor gamma t; SCID, severe combined immunodeficiency; SFMCs, synovial fluid mononuclear cells; SHP2, Src homology phosphatase 2; SNEDDS-TG, tangeretin in self-nano-emulsifying drug-delivery system; Tan IIA, Tanshinone IIA.

**Table 4:** The potential of multiple strategies for targeting lactate and its upstream and downstream signaling in IVDD.

Model type and sample sizes	Treatments	Mechanism of action	Main outcomes	References
Cellular Model: Primary human NP cells.	Intervention: IL-1β, Torin1, lactate. Dose & Route: In vitro: IL-1β (10 ng/mL, 24h); Torin1 (100 nM, 24h); L-lactate (50/100 mM, 24h). Safety alert: Torin1 (100 nM) protective; 10 μM toxic. Lactate toxic at 50/100 mM.	IL-1β: mTORC1 activity↑ (p-S6K↑, p-S6↑, p-4EBP1↑). Torin1 blocks mTORC1 and alters glucose metabolism. Exogenous lactate induces NPC damage.	In vitro: IL-1β: mTORC1 activation↑. Torin1 blocked apoptosis. Metabolomics: Torin1 altered glucose metabolism, lactic acid↓. Lactate: Cell viability↓, Apoptosis↑.	[125]
Human Observational: NP tissue (n = 24, grades II-V). Animal Model: Male SD rats, PIDD model (n = 6). Cellular Model: Primary rat NPCs.	Intervention: LVV-LOX, lactate, SC79, Akt mutants. Dose & Route: In vivo: Intra-disc LVV-LOX. In vitro: Lactate (2-10 mM, 4d); SC79; Akt mutants. Safety alert: No lentivirus toxicity. Lactate (6.10 mM) induced senescence and oxidative stress.	Lactic acid content↑ in degenerated tissue. Lactate: p-Akt↓, PI3K/Akt and Akt/Nrf2/HO-1 pathways↓.	Human Observational: Lactic acid content↑ with Pfirrmann grade. In vivo: LVV-LOX attenuated PIDD (MRI grade↓, DHI%↑, histology score↓); COL2↑, MMP9/13↓. In vitro: Lactate: Senescence and oxidative stress↑. LOX or SC79 reversed effects. Akt mutants reduced lactate effects.	[126]
Cellular Model: Primary human NP cells (grades I-II and IV-V).	Intervention: Genetic (*Sirt1*/*Ldha* OE/KD), c-Myc inhibitor. Dose & Route: In vitro: Lentiviral Ad-*Sirt1*, shRNA-*Sirt1*, shRNA-*Ldha*; c-Myc inhibitor. Safety alert: Standard procedures.	SIRT1 expression↓ in degeneration. SIRT1/c-Myc/LDHA axis promotes glycolysis. *Ldha* KD: Glycolytic ATP↓. c-Myc inhibitor: LDHA↓.	In vitro: *Sirt1* OE: Proliferation↑, apoptosis and senescence↓; Matrix↑. *Sirt1* KD had opposite effects. Seahorse: SIRT1↑, Glycolytic ATP↑; *Ldha* KD: Glycolytic ATP↓. *Ldha* KD mimicked *Sirt1* KD effects.	[127]
Animal Model: Male SD rats, PIDD model with lactate injection (n = 6). Cellular Models: Primary human/rat NPCs, human BMSCs.	Intervention: Novel delivery: LMGDNPs with BMSCs. Dose & Route: In vivo: Intra-discal co-injection of BMSC-loaded microspheres at 1 and 5 wks. In vitro: Co-culture with LMGDNPs and lactate (6 mM). Safety alert: LMGDNPs non-cytotoxic.	LMGDNPs consume extracellular lactate via LOX-MnO_2_. Reverse lactate-induced apoptosis (BAX↓, Bcl2↑). Alleviate autophagy blockade via lncRNA *Tgfb2-ot1*. Correct matrix imbalance.	In vivo: LMGDNPs: BMSC survival↑; Preserved NP hydration and structure, anabolic markers↑. In vitro: LMGDNPs protected NPCs from lactate-induced apoptosis. Autophagy or *Tgfb2-ot1* KD abolished protection.	[128]
Animal Model: Male SD rats, lactate-induced IDD model (n = 3 per time point). Cellular Model: Primary rat NPCs.	Intervention: Novel delivery: MS@MCL. Dose & Route: In vivo: Intra-discal MS@MCL 3 days post-induction. In vitro: Co-culture with MS@MCL and lactate (6 mM). Safety alert: MS@MCL non-cytotoxic and cytoprotective.	MS@MCL consumes lactate, generates O_2_, increases pH. Reduces lactate-induced inflammatory cytokines and catabolic enzymes. p53/MAPK pathways↓.	In vivo: MS@MCL: DHI%↑, Preserved NP hydration, improved histology score↑; COL2↑, IL-1β↓. In vitro: MS@MCL protected NPCs from lactate-induced death, intracellular ROS↓; Restored matrix proteins, inflammatory and catabolic gene expression↓.	[129]
Cellular Models: Primary bovine NP cells (n = 3). 5-layered 3D NPD model.	Intervention: Lactate, MCT1 inhibitor (AZD3965), and hypoxia mimic (CoCl_2_). Dose & Route: In vitro: L-lactate (0.5-20 mM, 10d); AZD3965; CoCl_2_. In 3D model: Pre-treatment with AZD3965. Safety alert: AZD3965 no cytotoxicity.	Lactate accumulates in 3D model. AZD3965: Intracellular lactate↓. Lactate + hypoxia: MMP-3↑. Lactate (20 mM): GAG↓. AZD3965 reverses lactate effects.	In vitro: 3D model: Chondrogenic markers and GAG diminished top to bottom; MMP-3↑. Lactate (20 mM): GAG↓, MMP-3↑. AZD3965 reversed lactate-induced GAG↓ and MMP-3↑. Lactate + CoCl_2_: MMP-3↑.	[130]
Human Observational: NP tissue (7 degenerative vs. 7 normal). Animal Model: SD rats, intra-discal lactate model (n = 4). Cellular Model: Primary human NPCs.	Intervention: Lactate, NLRP3 inhibitor (MCC950), ASIC inhibitors, ROS scavengers, NF-κB inhibitor (PDTC), siRNA. Dose & Route: In vitro: Lactate (6 mM, 24h); MCC950 (10 μM); Amiloride (100 μM); PcTx1 (20 nM); APETx2 (2 μM); NAC (10 mM); TEMPO (50 μM); PDTC (100 μM); siRNA (100 nM, 48h). In vivo: Intra-discal lactate (0-10 mM) ± Amiloride (100 μM). Safety alert: No overt toxicity. Inhibitor concentrations standard.	Lactate: *Asic1a/3* expression and activation↑; Ca^2+^ influx↑; ROS↑, NF-κB activation (p-IκBα, p-p65↑); NLRP3 inflammasome expression, assembly and activation↑; Casp-1 cleaves pro-IL-1β and GSDMD↑; IL-1β release and pyroptosis↑.	Human Observational: ASIC1a/3, NLRP3, Casp-1, IL-1β↑; COL2, ACAN↓ in degenerative tissue. In vitro: Lactate: Pyroptosis markers↑; Matrix↓, Catabolic enzymes↑. Inhibitors/siRNA reversed effects. In vivo: Lactate induced dose-dependent degeneration. Amiloride co-injection attenuated degeneration, inflammasome markers↓, matrix↑.	[131]
Human Data: NP tissue; bulk and scRNA-seq datasets. Animal Model: C57BL/6 mice, AF needle puncture-induced IVDD. Cellular Model: Primary human NPCs.	Intervention: IL-1β, CBX3 inhibitor (atosiban acetate). Dose & Route: In vitro: IL-1β (0-50 ng/mL, 24h); atosiban acetate (1-50 μM, 24h; 10 μM for assays). In vivo: i.p. atosiban acetate (0.14 mg/kg). Safety alert: 10 μM improved viability; 20/50 μM benefits reduced. No in vivo toxicity.	Pan-Kla↑ in human IVDD. CBX3 expression↑. Atosiban acetate binds/inhibits CBX3. Atosiban acetate: IL-1β-induced LDHA/PKM2 and Pan-Kla↓.	Human Observational: 6-hub LRG signature with IVDD severity. In vitro: Atosiban acetate reversed IL-1β-induced NPC degeneration: ACAN and COL2A1↑; ADAMTS5 and MMP-3↓. Inhibited IL-1β: upregulation of CBX3, LDHA, PKM2, and lactylation↑. In vivo: Atosiban acetate: Histological degeneration↓; Disc structure↑; ACAN↑, MMP-3↓.	[132]
Human Observational: NP tissue (n = 20, grades II-V); scRNA-seq dataset. Animal Model: Male C57BL/6 mice, needle puncture-induced IVDD (n = 5). Cellular Model: Primary human NPCs.	Intervention: Lactate, IL-1β, 2-DG, ferroptosis inhibitor, CHC, A485, siRNA, AAV9-si-*Ldha*. Dose & Route: In vitro: Lactate (10 mM); IL-1β (10 ng/mL); 2-DG (1 μM); inhibitors; siRNA. In vivo: Intradiscal AAV9-si-*Ldha*; i.p. 2-DG. Safety alert: AAV9 well-tolerated. 2-DG (1 μM) promoted proliferation.	scRNA-seq: Glycolysis score↑; OXPHOS score↓ in IVDD. Lactate: H3K18la↑, Enriches at *Acsl4* promoter, *Acsl4* expression and lactylates ACSL4 protein (K412) ↑; SIRT3↓ via miR-708-5p.	Human Observational: HK2, G6PD, LDHA, lactate↑ with Pfirrmann grade. In vitro: Lactate: Ferroptosis markers↑; Viability and mitochondrial potential↓; Anabolic markers↓; Catabolic markers↑. Inhibitors of lactate production, transport, ACSL4 reversed these effects. In vivo: AAV9-si-*Ldha* and 2-DG attenuated IVDD: disc height↑, histopathology↓; lactylation/4-HNE/ACSL4↓, ACAN expression↑.	[133]

**Abbreviations:** 4-HNE, 4-hydroxynonenal; APETx2, *Anthopleura elegantissima* toxin 2; CBX3, chromobox homolog 3; CHC, α-cyano-4-hydroxycinnamate; lncRNA, long non-coding RNA; LMGDNPs, LOX-MnO_2_ nanozyme-loaded glucose-rich nucleus pulposus dECM hydrogel-based nano/microparticles; LOX-MnO_2_, -lactate oxidase-manganese dioxide nanozyme; LVV-LOX, lentiviral vectors carrying the lactate oxidase gene; MCC950, a NLRP3 inhibitor; MnO_2_, manganese dioxide; MRI, magnetic resonance imaging; MS@MCL, microsphere encapsulated MnO_2_-Chitosan-Lactate Oxidase nanozyme; NAC, N-acetylcysteine; p-4EBP1, phosphorylated 4E-binding protein 1; p53, tumor protein p53; PcTx1, psalmotoxin 1; PDTC, pyrrolidine dithiocarbamate; p-IκBα, phosphorylated inhibitor of nuclear factor kappa B alpha; PIDD, puncture-induced intervertebral disc degeneration; p-p65, phosphorylated nuclear factor kappa B p65 subunit; p-S6K, phosphorylated S6 kinase; scRNA-seq, single-cell RNA sequencing; TEMPO, (2,2,6,6-tetramethylpiperidin-1-yl) oxyl; Tgfb2-ot1, transforming growth factor beta 2 overlapping transcript 1.

**Table 5:** The potential of multiple strategies for targeting lactate and its upstream and downstream signaling in OS.

Model type and sample sizes	Treatments	Mechanism of action	Main outcomes	References
Human Observational: Serum, paired OS/peri-OS tissue, OS tissue, public datasets. Animal Model: BALB/c mice, K7M2 xenograft. Cellular Models: Human/murine OS cells, THP-1 macrophages.	Intervention: In vitro: 2-DG, *Mif* KO, rMIF. In vivo: *Mif* KO cells, 4-IPP, clodronate liposomes, anti-PD-1. Dose & Route: In vitro: 2-DG. In vivo: i.p. 4-IPP (10 mg/kg, q3d); i.v. clodronate liposomes; i.p. anti-PD-1. Safety alert: 4-IPP no overt toxicity. 2-DG standard.	Lactate and H3K9la↑ in OS. Lactate: H3K9la enrichment at *Mif* promoter↑; MIF expression↑ in OS cells. 2-DG: MIF↓, H3K9la↓. MIF: anti-inflammatory TAM polarization↑, TAM chemotaxis↓. MIF-polarized TAMs: CXCL9/CXCL10↓; CD8^+^ T cell function↓.	Human Observational: Serum/tumor lactate↑ correlates with prognosis. High-risk lactate signature: Immune score↓; Anti-inflammatory TAMs↑. High MIF: Survival↓, Anti-inflammatory TAMs↑; CD8^+^ T cells↓. In vivo: *Mif* KO or 4-IPP: Tumor growth↓; TAM and CD8^+^ T cell infiltration↑; Anti-inflammatory TAMs↓. Macrophage depletion attenuates 4-IPP. 4-IPP^+^ anti-PD-1: Synergistic growth inhibition; CD8^+^ cells and CXCL9/CXCL10↑. In vitro: High-risk OS cells secrete MIF: Anti-inflammatory macrophage polarization↑; Migration↓. *Mif* KO or 2-DG: CXCL9/CXCL10↑; CD8^+^ T cell cytotoxicity↑.	[134]
Animal Model: BALB/c (nu/nu) mice, HOS xenograft (n = 6). Cellular Models: Human OS cell lines.	Intervention: In vitro: FX11, shRNA-*Ldha*, 2-DG, c-Myc inhibitor, HIF-1α inhibitor. In vivo: sh-Ctrl or sh-*Ldha* HOS cells. Dose & Route: In vitro: FX11, 2-DG, c-Myc inhibitor, HIF-1α inhibitor. Safety alert: FX11 specific LDHA inhibitor. No overt toxicity.	LDHA↑ in OS cell. LDHA inhibition (FX11) or silencing: LDHA activity and lactate↓. *Ldha* silencing: ECAR↓, Glycolytic capacity↓; Glucose consumption↓. LDHA effects blocked by 2-DG. c-Myc or HIF-1α inhibition: LDHA↓.	In vivo: *Ldha* silencing: Tumor size/weight↓; PCNA^+^ cells↓; TUNEL^+^ cells↑; Caspase-3/7 activity↑. In vitro: FX11 or *Ldha* KD: Proliferation and viability↓; Invasion↓, MMP-9↓. 2-DG co-treatment blocks effects. c-Myc/HIF-1α inhibition: LDHA↓.	[135]
Human Observational: 63 OS tissues and controls; metastatic vs. non-metastatic OS. Animal Model: BALB/c nude mice, orthotopic induced lung metastasis (143B-luc). Cellular Models: OS cell lines 143B, HOS.	Intervention: In vitro: shRNA-*Kdm6b*, shRNA-*Ldha*, GSK-J4, *Ldha* OE. In vivo: Orthotopic sh-*kdm6b* cells; i.p. GSK-J4 (5 mg/kg). Dose & Route: In vitro: GSK-J4 (0.8 μM). In vivo: GSK-J4 (5 mg/kg, i.p.). Safety alert: GSK-J4 selective KDM6B inhibitor. No overt toxicity.	*Kdm6b* KD: H3K27me3 at *Ldha* promoter↑; *Ldha* expression↓; LDH activity↓; Glucose uptake↓; Lactate production↓. *Ldha* OE reverses glycolytic inhibition. Upstream c-Myc or HIF-1α inhibition: LDHA↓.	Human Observational: *Kdm6b* mRNA↑ in OS tissues and metastatic OS. H3K27me3 protein↓ in metastatic OS. High KDM6B correlates with poor survival. In vitro: *Kdm6b* KD or GSK-J4: Migration and invasion↓. *Ldha* KD similar. *Ldha* OE reverses *Kdm6b* KD migration effects. In vivo: *Kdm6b* KD or GSK-J4 treatment: Lung metastasis↓.	[136]
Human Observational: OS TMA (n = 71 OS, 20 normal), TCGA. Animal Model: BALB/c nude mice, 143B xenograft. Cellular Models: OS cell lines 143B, U2OS; HEK-293T.	Intervention: In vitro: siRNA/shRNA-*Nat10*, siRNA- *Ythdc1*, Lenti-*Ythdc1*, Actinomycin D, Cycloheximide. In vivo: 143B cells with sh-*Nat10* or sh-*Nat10*+Lenti-*Ythdc1*. Dose & Route: In vitro: Actinomycin D (10 μg/mL), Cycloheximide. Safety alert: Standard. No overt toxicity.	NAT10/ac4C: global m6A↓. *Nat10* KD: m6A↑. NAT10 (via ac4C) regulates YTHDC1 stability. YTHDC1 binds *Pfkm* and *Ldha* mRNAs: stability↑. *Ythdc1* OE partially abrogates *Nat10* KD.	Human Observational: NAT10 protein↑ in OS. High NAT10 correlates with poor survival. In vitro: *Nat10* or *Ythdc1* KD: OS cell proliferation, migration, invasion↓; Lactate↓, Pyruvate↓, Glucose uptake↑; LDHA and PFKM↓. *Nat10* KD: YTHDC1↓. YTHDC1 binds and stabilizes *Ldha* and *Pfkm* mRNA. In vivo: *Nat10* KD: Tumor growth↓. *Ythdc1* OE partially restores growth. Sh-*Nat10* tumors: PFKM/LDHA↓, Partially reversed by *Ythdc1* OE.	[137]
Human Observational: 50 paired OS/adjacent tissues. Cellular Models: Human OS cell lines, normal OBs.	Intervention: In vitro: miR-323a-3p mimics, antagomir, control miRNA. Dose & Route: In vitro: Transfection. Safety alert: Standard transfection. No cytotoxicity.	miR-323a-3p binds LDHA: *Ldha*↓. miR-323a-3p inhibits glycolysis: Glucose consumption↓; Lactate↓. LDHA inversely correlates with miR-323a-3p.	Human Observational: miR-323a-3p expression↓ in OS tissues and metastatic OS. *Ldha* mRNA↑ in OS tissues. In vitro: miR-323a-3p OE: Viability↓, Colony formation↓; Apoptosis↑; Glucose consumption↓; Lactate↓. miR-323a-3p KD opposite effects.	[138]
Human Observational: 33 paired OS/adjacent tissues. Animal Model: BALB/c nude mice, U2OS xenograft (n = 6). Cellular Models: Human OS cell lines, normal OBs.	Intervention: In vitro: siRNA/shRNA-circ_0000376, miR-577 mimic/inhibitor, pcDNA-*Hk2*/*Ldha*. In vivo: U2OS cells with sh-circ_0000376 or sh-NC. Dose & Route: Standard transfection/transduction. Safety alert: Standard. No overt toxicity.	Circ_0000376 sponges miR-577: miR-577↓. miR-577 targets HK2 and LDHA, represses expression. Circ_0000376/miR-577 axis: HK2 and LDHA↑. Upregulated HK2/LDHA: Glucose consumption↑; Lactate↑, ATP/ADP↑, ECAR↑, OCR↓.	Human Observational: Circ_0000376↑, miR-577↓, *Hk2* and *Ldha* mRNA↑ in OS. Circ_0000376 negatively correlates with miR-577; miR-577 negatively with HK2/LDHA. In vitro: Circ_0000376 KD or miR-577 OE: Viability, colony formation, apoptosis, EdU^+^ rate↓, Invasion↓, MMP9↓; Glycolysis↓ (glucose consumption↓, lactate↓, ATP/ADP↓, ECAR↓, OCR↑). *Hk2/Ldha* OE reverses miR-577 effects. In vivo: Circ_0000376 KD: Tumor growth↓. Tumors: miR-577↑, HK2/LDHA protein↓, Ki67^+^ cells↓.	[139]
Human Observational: 29 paired OS/adjacent tissues. Animal Model: BALB/c nude mice, U2OS xenograft (n = 8). Cellular Models: Human OS cell lines, normal OBs.	Intervention: In vitro: siRNA/shRNA-circ-CNST, miR-578 mimic/inhibitor, pcDNA-*Ldha*/*Pdk1*. In vivo: U2OS cells with sh-circ-CNST or sh-NC. Dose & Route: Standard transfection/transduction. Safety alert: Standard. No overt toxicity.	Circ-CNST sponges miR-578: miR-578↓. miR-578 targets LDHA and PDK1, represses expression. Circ-CNST/miR-578 axis: LDHA and PDK1↑; Glucose consumption↑; Lactate↑, ATP/ADP↑.	Human Observational: Circ-CNST↑, miR-578↓, *Ldha*/*Pdk1* mRNA↑ in OS tissues. High circ-CNST/LDHA/PDK1 or low miR-578 correlates with poor survival, advanced stage, lymph node metastasis. In vitro: Circ-CNST KD or miR-578 OE: OS cell proliferation, migration, invasion↓; Apoptosis↑; Glycolysis↓ (glucose consumption↓, lactate↓, ATP/ADP↓). *Ldha*/*Pdk1* OE reverses effects. In vivo: Circ-CNST KD↓, Tumor growth↓. Tumors show lower circ-CNST levels.	[140]
Human Observational: 30 paired OS/adjacent tissues. Animal Model: BALB/c nude mice, cisplatin-resistant Saos-2 xenograft. Cellular Models: OS cell lines Saos-2, MG-63 and cisplatin-resistant derivatives.	Intervention: In vitro: miR-329-3p precursor, cisplatin (CDDP), 2-DG, LDHA plasmid. In vivo: Xenograft with miR-329-3p OE. Dose & Route: In vitro: Cisplatin (2.5-80 μg/mL), 2-DG (2 mM). Safety alert: Cisplatin and 2-DG are known agents.	miR-329-3p binds LDHA: LDHA↓. miR-329-3p inhibits glycolysis: Glucose uptake↓; Lactate production↓. miR-329-3p and *Ldha* mRNA negatively correlate. CDDP-R OS cells: miR-329-3p↓, Glycolysis↑ (glucose uptake↑, lactate↑), *Glut1*, *Hk2*, *Pkm2*, *Ldha* mRNA↑).	Human Observational: miR-329-3p expression↓ in OS tissues. In vitro: miR-329-3p OE: OS cell proliferation↓; Sensitizes CDDP-R cells to cisplatin (survival↓). LDHA restoration reverses sensitization. miR-329-3p OE: Glucose uptake and lactate↓; LDHA reverses. 2-DG sensitizes OS cells to cisplatin. Low-toxic cisplatin↓ miR-329-3p; High/apoptotic concentration↑ it. miR-329-3p↓ in CDDP-R vs. parental cells. In vivo: miR-329-3p OE: Tumor growth↓.	[141]
Human Observational: OS TMA (n = 61). Animal Models: BALB/c nude mice, subcutaneous and orthotopic xenograft. Cellular Models: Human OS cell lines, drug-resistant lines, primary cultures.	Intervention: In vitro: shRNA-*Mct1*, CHC (MCT1 inhibitor), ADM, p65 OE. In vivo: i.p. CHC (25 μmol daily) ± i.p. ADM (6 mg/kg weekly). Dose & Route: In vitro: CHC (0-20 nM; 5-10 nM), ADM. In vivo: CHC (25 μmol, i.p., daily), ADM (6 mg/kg, i.p., weekly). Safety alert: CHC no side effects. ADM caused weight loss.	MCT1 expression↑ in OS. MCT1 inhibition: p65 protein levels and phosphorylation (NF-κB pathway inhibition) ↓. p65 OE partially reverses anti-proliferative/anti-invasive effects of CHC.	Human Observational: High MCT1 expression in 67.3% of OS, correlates with worse overall survival. In vitro: MCT1 KD or CHC: OS cell proliferation↓; Migration and invasion↓. CHC: ADM cytotoxicity↑. In vivo: CHC monotherapy: Tumor growth↓. CHC + ADM superior growth inhibition vs. monotherapies.	[142]
Human Observational: 80 primary/recurrent OS tissues, 65 paired OS/adjacent tissues, other sarcoma tissues, TCGA/TARGET databases. Animal Model: BALB/c nude mice, subcutaneous xenograft and tail-vein lung metastasis. Cellular Models: Human OS cell lines, normal BMSCs.	Intervention: In vitro: Lactate, *Gpr81*/*β-Arrestin1/2* OE/KD/KO, Pertussis toxin, Endothall (PP2A inhibitor). In vivo: i.p. Endothall (3 mg/kg q2d) ± Cisplatin (5 mg/kg q2d); *Gpr81*-KO 143B cells. Dose & Route: In vitro: In vitro: Lactate (10 mM), Endothall. In vivo: Endothall (3 mg/kg, i.p., q2d), Cisplatin (5 mg/kg, i.p., q2d). Safety alert: Standard. No overt toxicity from Endothall.	Lactate activates GPR81. Lactate- GPR81 signals via β-Arrestin2 to recruit PP2A. Dephosphorylated STAT1/2: Transcriptional activity↓; Anti-oncogenic genes↓. PP2A inhibitor Endothall: STAT1/2 dephosphorylation↓.	Human Observational: GPR81 expression↑ in OS. High GPR81 correlates with poor survival, advanced stage, recurrence, metastasis, mortality. In vitro: *Gpr81* OE or lactate (10 mM): OS cell proliferation, colony formation, migration, invasion, G2-M phase↑. *Gpr81* or *β-Arrestin2* KO, or Endothall, reverse lactate effects (STAT1/2 dephosphorylation↓, pro-tumor phenotypes↓, apoptosis↑). *β-Arrestin1* KO or Pertussis toxin no reverse. In vivo: *Gpr81* KO or Endothall: Subcutaneous tumor growth↓; Lung metastasis↓. Endothall + Cisplatin synergy. *Gpr81* KO or Endothall: p-STAT1/2↑ in tumors. *Gpr81*^+^ cells: Ki67^+^ but p-STAT1/2 low.	[143]
Cellular Model: UMR106 rat OS cell line (n = 5-9).	Intervention: In vitro: L-lactic acid (1-25 mM), Nala (1-25 mM), Formic acid (22.8 mM), Acetic acid (10 mM), NF-κB inhibitors (Withaferin A 500 nM, Wogonin 100 μM). Priming with 1,25(OH)_2_D_3_. Dose & Route: In vitro: 24h treatment with acids/inhibitors after 24h priming with calcitriol (10 nM). Safety alert: Standard. No specific cytotoxicity data.	Lactic acid-induced *Fgf23* upregulation attenuated by NF-κB inhibitors. Acids that lower pH (lactic, formic, acetic): *Fgf23*↑. Nala: *Fgf23*↑, indicating pH-independent lactate ion effect.	In vitro: Lactic acid dose-dependently *Fgf2*3 mRNA↑. 25 mM lactic acid: C-terminal FGF23 protein secretion↑. Acids that lower pH upregulate *Fgf23*.	[144]
Human Observational: 56 paired OS/adjacent tissues. Cellular Models: Human OS cell lines, normal OBs.	Intervention: In vitro: shRNA-*Ldhb*, shRNA-*Fus*, pcDNA3.1-*Fus*, pcDNA3.1-Ldhb, miR-141-3p mimics. Dose & Route: Standard transfection/transduction. Safety alert: Standard. No overt toxicity.	miR-141-3p targets FUS: *Fus* expression↓. FUS binds *Ldhb* mRNA: stability↑. miR-141-3p↓: FUS↑, *Ldhb* mRNA stability and expression↑. miR-141-3p OE: FUS and LDHB↓. *Fus* OE: Reverses miR-141-3p-mediated LDHB↓.	Human Observational: *Ldhb* and *Fus* mRNA↑ in OS; positively correlated. miR-141-3p↓ in OS cell lines. In vitro: *Ldhb* KD or miR-141-3p OE: Proliferation↓, Apoptosis↑, Invasion↓, Migration-related proteins and EMT markers↓. *Ldhb* OE reverses miR-141-3p effects. *Fus* OE: *Ldhb* mRNA stability↑; co-OE miR-141-3p impairs.	[145]

**Abbreviations**: 4-IPP, 4-iodo-6-phenylpyrimidine; ac4C, N4-acetylcytidine; ADM, adriamycin; Antagomir, antisense oligonucleotide against microRNA; CDDP, cisplatin; EdU, 5-ethynyl-2′-deoxyuridine; EMT, epithelial-mesenchymal transition; FUS, fused in sarcoma; GSK-J4, a KDM6B inhibitor; m6A, N6-methyladenosine; PCNA, proliferating cell nuclear antigen; PD-1, programmed cell death protein 1; PFKM, PFK, muscle type; TAMs, tumor-associated macrophages; YTHDC1, YTH domain containing 1

### 3.1 Lactate in the pathogenesis and therapy of OP

OP is characterized by reduced bone mass and microarchitectural deterioration, fundamentally representing an imbalance between bone resorption and formation [146]. In contrast to its role in other skeletal disorders, where it functions as a catabolic mediator, in the pathological process of OP, lactate primarily serves as a physiological signaling molecule that promotes bone formation. Its systemic reduction, in combination with impaired local signaling, collectively defines the metabolic hallmark of this disease. Lactate signals have been demonstrated to mediate the osteogenic effects of mechanical stimuli and to coordinate metabolic communication between bone and peripheral organs, such as muscle and blood vessels.

#### 3.1.1 Targeting lactate metabolism in OP

The metabolic landscape of bone is profoundly altered in OP, with lactate serving as a key indicator and mediator of this dysfunction. The finding of significantly lower serum lactate levels in OP patients suggests a potential disruption to metabolic flux on a global or local scale [21], which may be linked to reduced physical exercise, altered muscle-bone communication, or an inappropriate compensatory mechanism. This systemic change is mirrored and amplified at the tissue level by pathological metabolic reprogramming of bone marrow cells. For instance, the release of dysfunctional mitochondria from macrophages in the osteoporotic niche demonstrates how immune-metabolic dysfunction can trigger a compensatory glycolytic shift, thereby increasing lactate production [147]. The targeting of this metabolic vulnerability has been demonstrated to be a promising therapeutic approach, as evidenced by the efficacy of visomitin, which has been shown to inhibit osteoclastogenesis and bone resorption by suppressing STAT3-dependent LDHB expression and lactate production [89]. The utilization of plant extracts, such as *Lepidium sativum* extract, has been demonstrated to mitigate bone loss, enhance bone biomechanical properties, and regulate serum LDH activity and bone metabolism markers and signaling, such as C-telopeptide of type I collagen (CTX), TRAP, and RANK/OPG in ovariectomized (OVX) rats [90] (Table 1). Furthermore, the field of neuroendocrine regulation intersects with that of lactate metabolism. Recent studies have indicated a potential correlation between the protective effects of melatonin against OP and lactate metabolism in models involving lipopolysaccharide (LPS) and cadmium. The process functions are related to the reduction of LDH release and lactate production, the suppression of mitophagy-induced mitochondrial ROS (mtROS) generation, and N-terminal fragment of Gasdermin D (GSDMD)-mediated OB pyroptosis, among others [93,94,148] (Table 1). It is evident that abnormal lactate metabolism constitutes a pivotal factor in the onset and progression of OP. These processes not only serve as novel biomarkers for disease diagnosis but also represent highly promising therapeutic targets by regulating bone metabolism.

#### 3.1.2 Targeting lactate shuttle in OP

The lactate shuttle is a critical determinant of the metabolic fate of bone cells in OP, and thus represents a direct target for intervention. Pharmacologically, the systemic administration of the MCT inhibitor AZD3965 in OVX mice resulted in a significant increase in bone mineral density (BMD), thereby enhancing trabecular bone mass, thickness, and connectivity. From a mechanistic perspective, AZD3965 exerts its inhibitory effect on OC formation and resorptive function in vitro by impeding lactate export, resulting in the accumulation of intracellular lactate and subsequent downregulation of the pro-resorptive NF-κB/mitogen-activated protein kinase (MAPK) signaling [95]. This principle is also applicable to physiological regulation. The exercise-induced elevation of lactate, a pivotal osteogenic mediator, underlies the bone-protective effects of high-intensity interval training (HIIT) in OVX mice. The anabolic effect of HIIT is lactate-dependent, as evidenced by the ability to be abolished by MCT inhibition and the capacity to be faithfully mimicked by exogenous lactate administration [91] (Table 1). In addition to its role in bone cell-autonomous regulation, lactate has been shown to integrate the regulation of multiple tissues in the maintenance of skeletal homeostasis. The delivery of LDHA to BMSCs by skeletal muscle-derived extracellular vesicles has been shown to enhance their glycolytic capacity and lactate production, thereby promoting osteogenesis and protecting against disuse OP [149]. Paracrine lactate from endothelial cells has also been demonstrated to be imperative for the maintenance of bone formation. The endothelial-specific deletion of the *Pkm2* gene has been demonstrated to reduce lactate secretion, to impair osteogenic differentiation, and to exacerbate bone loss [21]. Evidence has demonstrated the existence of immunometabolic crosstalk within the adipose-bone axis. This crosstalk has the capacity to induce lactate accumulation within the bone marrow macrophages. This lactate overload has been demonstrated to disrupt the lysosomal ATP6V0d2, thereby suppressing lipolysis-dependent bioenergetics and osteoclastogenesis, thus forming a self-limiting circuit that constrains inflammation-driven bone loss [92] (Table 1). In summary, lactate has been demonstrated to regulate the coordination of skeletal muscle, the vascular system, adipose tissue and bone. This finding underscores the therapeutic potential of targeting lactate transport pathways to achieve synergistic regulation of bone metabolism.

#### 3.1.3 Targeting lactate receptor in OP

GPR81 has been identified as playing a dual role in the anti-osteoporotic effects of exercise-induced lactate, both promoting OB differentiation from pre-OBs and simultaneously inhibiting OC formation. Specifically, in OBs, GPR81 activation has been shown to stimulate the canonical Wnt/β-catenin pathway, thereby promoting osteogenic differentiation. In contrast, in OCs, GPR81 activation has been shown to inhibit cell differentiation by activating the TGF-β-activated kinase-1 (TAK1)-p65 signaling axis. The absence of these critical effects in *Gpr81*-deficient cells provides unequivocal confirmation of the indispensable role of the receptor. In vivo experiments in OVX mice further demonstrated that both HIIT and direct exogenous lactate administration effectively slowed bone resorption, enhanced bone formation, and mitigated bone loss [96] (Table 1). Collectively, these findings elucidate that the GPR81 serves as the primary sensor, translating elevated lactate concentrations into anabolic and anti-catabolic cellular responses in bone.

#### 3.1.4 Targeting lactylation modification in OP

Lactylation functions as both a diagnostic biomarker, with proteins such as cysteine and glycinerich protein 2 and far upstream element-binding protein 1 indicating early risk, and as a functional regulator that directs osteogenic differentiation in BMSCs through specific gene signatures. Furthermore, the observation that shared lactylation-related pathways exist between OP and chronic kidney disease provides a molecular basis for their frequent co-morbidity [[150], [151], [152]]. Mechanistically, lactylation orchestrates osteogenesis through multiple interconnected pathways. Anabolic signals, such as those derived from a small leucine-rich proteoglycan lumican, have been shown to promote osteogenic differentiation of BMSCs by enhancing glycolytic flux and lactate production. This, in turn, has been demonstrated to drive H3K18la at osteogenic gene promoters, thereby establishing a direct link from metabolism to epigenetic activation [153]. The significance of this pathway is further substantiated by the observation that physical exercise or exogenous lactate administration elevates blood lactate levels, consequently augmenting H3K18la enrichment and osteogenic gene expression in BMSCs [21] (Table 1). In addition to histone modification, lactylation of the small heat shock protein αB-crystallin (CRYAB) has been demonstrated to promote osteogenic differentiation of BMSCs by regulating the ferritin heavy chain 1 (FTH1), thus identifying lactylation-dependent control of ferroptosis as a critical downstream mechanism [154]. Furthermore, lactylation has been demonstrated to contribute to immunometabolic dysregulation within the osteoporotic bone marrow niche. The lactylation-related gene AKR1A1 has been observed to undergo significant upregulation and lactylation within monocytes and macrophages. In these cells, it has been demonstrated to participate in the secreted phosphoprotein 1 (SPP1)-cluster of differentiation 44 (CD44) signaling pathway, thereby mediating metabolic reprogramming and shaping a pro-resorptive immune microenvironment. This process establishes to establish a link between altered metabolism and immune-mediated bone loss [155]. The multifaceted role of lactylation is thus established as both a compelling diagnostic biomarker and a promising therapeutic target for OP.

### 3.2 Lactate in the pathogenesis and therapy of OA

OA is a progressive degenerative joint disorder characterized by articular cartilage erosion, subchondral bone sclerosis, and synovitis, driven by a convergence of biomechanical stress, chronic low-grade inflammation, and metabolic dysregulation [156]. In this process, lactate evolves from a mere metabolic byproduct into the core engine driving this cycle. The overproduction of lactate by diseased joint cells has been shown to result in the acidification of the local microenvironment. In addition, this has been demonstrated to result in the continuous amplification of inflammatory responses and matrix degradation signals through the activation of specific sensory pathways and epigenetic reprogramming. This dynamic, if left unaddressed, is likely to perpetuate a cycle of joint destruction.

#### 3.2.1 Targeting lactate metabolism in OA

The OA joint microenvironment, characterized by hypoxia and inflammation, has been shown to trigger a pathogenic metabolic reprogramming in both chondrocytes and synovial cells. This reprogramming is characterized by a transition from the basal glycolysis observed in healthy cartilage to a hyperactive, inducible “glycolytic burst”. The process is driven by pro-inflammatory cytokines (e.g., IL-1β) via the NF-κB pathway and involves the upregulation of key enzymes such as LDHA and PKM2. The consequence of this process is not only an increase in energy production, but also a substantial and unregulated overproduction of lactate [23,75,[157], [158], [159]]. The transformative potential of this metabolic axis is further demonstrated by the identification of lactate metabolism-related genes, such as solute carrier family 2, member 1 (SLC2A1) and NADH dehydrogenase (ubiquinone) 1β subcomplex, 9, 22 kDa (NDUFB9), both of which are recognized as potential diagnostic biomarkers [160]. Furthermore, the intra-articular injection of lactate has been demonstrated to induce catabolic and pro-inflammatory responses in chondrocytes, concomitantly activating the arginase 2 (ARG2)-mTOR/ribosomal protein S6 kinase B1 (S6K1)/eukaryotic translation initiation factor 4B (eIF4B) and the p53/p21 pathways, as well as promoting senescence-associated secretory phenotype (SASP) expression in fibroblast-like synoviocytes (FLSs). This process has been shown to expedite the progression of OA [23,97]. Recent studies have identified specific molecular drivers of this glycolytic shift. For instance, the follistatin-like 1 (FSTL1) and the ubiquitin-specific protease 32 (USP32) have been demonstrated to significantly enhance the expression of key glycolytic enzymes in chondrocytes, thereby inducing lactate accumulation, which in turn fuels subsequent pathological processes [104,161]. However, other studies have indicated a more complex role, as expressions of LDHA and LDHC were found to be significantly downregulated in knee OA chondrocytes, and their restoration mitigated chondrocyte dysfunction [162]. Moreover, it has been demonstrated that L-lactate itself has the capacity to pharmacologically block the activation of mitochondrial antiviral signaling protein (MAVS). This process has been shown to attenuate NF-κB-dependent expression of MMP-3/13 and OA progression, thereby revealing a direct anti-inflammatory role of lactate [98], suggesting disease- or context-specific metabolic adaptations.

From a therapeutic standpoint, the targeting of lactate production emerges as a viable strategy. Pharmacological inhibition of LDHA using compounds such as GSK2837808A, FX11, or oxamate has been shown to attenuate OA progression by mitigating ROS overproduction, restoring energy balance, and reducing catabolic enzyme expression [72,99,100]. Furthermore, natural compounds (e.g., 5-hydroxymethylfurfural [5-HMF]) and electroacupuncture therapy modulate the GLUT1/HK1/LDHA and GLUT1/PKM2/LDHA axes, reducing cartilage hypoxia and lactate accumulation to protect joint integrity [101,102] (Table 2). Recent advancements in nanomedicine have resulted in the development of a LOX-based nanodrug delivery system guided by a hydrogel (HG-p-LOX). These systems have been engineered to remodel the metabolic microenvironment by specifically targeting glycolysis-mediated lactate production in chondrocytes. This approach provides a sustained therapeutic strategy for cartilage repair [163]. Convergent interventions have been demonstrated to emphasize lactate metabolism as a pivotal therapeutic target in OA.

#### 3.2.2 Targeting lactate shuttle in OA

Although potent anti-inflammatory agents such as dexamethasone (Dex) are mainstays for managing synovitis, their chronic use is paradoxically associated with cartilage deterioration. Mechanistic insight reveals that Dex epigenetically silences the promoter of the *Mct4* gene, a major conduit for lactate export, by reducing histone H3 lysine 4 trimethylation (H3K4me3). This transcriptional repression has been shown to result in a critical deficit in MCT4 expression. Consequently, impaired lactate efflux forces an abnormal intracellular accumulation of lactate, disrupting the metabolic gradient. The process of lactate overload has been shown to induce a detrimental cascade, resulting in apoptosis of the chondrocyte, mitochondrial dysfunction and elevated oxidative stress. These mechanisms have been proven to cause direct damage to cartilage tissue [103] (Table 2). This mechanism provides a compelling metabolic explanation for the well-documented cartilage-harming side effects and the limited long-term structural efficacy of intra-articular glucocorticoids in OA.

#### 3.2.3 Targeting lactate receptor in OA

Within the cellular environment, the interaction of lactate with GPR81 in chondrocytes serves as a catalyst for the phosphoinositide 3-kinase (PI3K)/Akt signaling pathway, ultimately resulting in the upregulation of NADPH oxidase 4 (NOX4). This cascade has been demonstrated to induce a significant overproduction of ROS, which subsequently stimulates the expression of key catabolic enzymes, including MMP-3/13, and ADAMTS-4. Additionally, it has been observed to stimulate the production of pro-inflammatory cytokines and chemokines, such as IL-6, C-C motif chemokine ligand 3 (CCL3), and CCL4. The net effect of these changes is a profound shift in the phenotype of the cells, with the inhibition of anabolic collagen synthesis coupled with the induction of cellular hypertrophy and senescence, collectively accelerating the degradation of the ECM [23] (Table 2). Consequently, the lactate-GPR81 axis fulfils a pivotal role as a metabolic sensor and signal transducer within the OA joint, highlighting GPR81 as a potential therapeutic target for intercepting this metabolically driven pathological communication.

#### 3.2.4 Targeting lactylation modification in OA

Integrative bioinformatics approaches have identified a panel of seven lactylation-related genes (LRGs) with high diagnostic value for OA [164]. These genes have the potential to influence the development and progression of OA by regulating immune responses. From a functional standpoint, histone lactylation exerts a significant regulatory influence on transcription processes within the joint. For instance, histone H4 lysine 12 lactylation (H4K12la) in chondrocytes have been demonstrated to promote the transcription of pro-inflammatory genes and reinforce a pathological positive feedback loop by sustaining PFKFB3-driven glycolysis and inflammation [105]. The interruption of this loop is a therapeutic possibility that has been demonstrated to be effective via the utilization of the natural compound known as songorine. In fibrocartilage cells, the induction of glycolytic flux and lactate accumulation by FSTL1 has been identified as a pivotal regulatory mechanism that activates H3K18la. This, in turn, facilitates the transcription of genes associated with fibrosis, thereby contributing to the perpetuation of a fibrotic phenotype [104]. In addition, LDHB-mediated H3K18la has been shown to epigenetically enhance the expression of acyl-CoA synthetase long-chain family member 4 (ACSL4), thereby promoting ferroptosis, a form of regulated cell death implicated in cartilage degradation [106]. In contrast, LDHA in BMSC-exosomes (BMSC-Exos) has been shown to promote BMP7 expression via H3K18la, which in turn has been demonstrated to facilitate tendon-bone healing following arthroscopic ACL reconstruction (ACLR) [107]. Furthermore, H3Kla can be modulated to alter disease outcomes, as evidenced by nanodrug systems (e.g., HG-p-LOX) that ameliorate OA partly by modifying histone lactylation and subsequent inflammatory gene transcription [163]. It is evident that the function of histone marking is context-dependent.

In the specific context of synovial macrophages exposed to hyperglycemic conditions, the augmentation of glycolysis leads to the substantial accumulation of lactate, ultimately culminating in the specific lactylation of the CD11b protein at K575. This modification has been demonstrated to modify the conformation of CD11b, thereby compromising its capacity for efferocytosis. The resulting defect in immune resolution has been shown to exacerbate synovial inflammation and accelerate OA progression [165]. In the case of the cell line, the process of lactylation at the K6 site of UDP-glucose dehydrogenase (UGDH) is stimulated by lactate. It has been demonstrated that this alteration hinders the interaction between UGDH and STAT1, consequently activating the MAPK kinase 8 (MAP3K8)-MAPK pathway and promoting ECM degradation. The process has been demonstrated to be pharmacologically reversible by the p300 inhibitor A485 [108] (Table 2). Collectively, these findings indicate that lactylation can interpret the metabolic state of joints (namely elevated lactate levels) as a specific pathogenic outcome, and this outcome exhibits cell-type dependency.

Recent studies have further elucidated the complexity and therapeutic potential of targeting lactylation. The transportation of OC-derived lactate is facilitated by MCT1/MCT4, which is then taken up by OBs. In turn, this process activates H4K12la, thereby driving osteogenic programmes. This process has been demonstrated to contribute to subchondral sclerosis, a condition that has been shown to be alleviated by MCT inhibition [166]. The functional outcome of lactylation is highly site-specific. While H4K12la and H3K18la frequently correlate with pathological onset, glycolytic lactate-driven histone H3 lysine 56 lactylation (H3K56la) has been shown to promote COL2 synthesis in chondrocytes via HIF-1α, indicating a protective role in cartilage homeostasis [167]. Beyond direct modulation, lactylation levels can be regulated upstream by inhibiting lactate production. For instance, coniferyl aldehyde, a constituent of ginger-processed Eucommiae Cortex, has been shown to ameliorate OA by inhibiting aldolase A (ALDOA), thereby reducing lactate and consequent histone H3 lysine 23 lactylation (H3K23la) levels [168]. Collectively, these findings emphasize the role of lactylation as a pivotal mediator in intercellular metabolic crosstalk within the joint.

### 3.3 Lactate in the pathogenesis and therapy of RA

RA is a chronic systemic autoimmune disorder characterized by synovial inflammation, anti-citrullinated protein antibody production, and progressive joint destruction, driven by dysregulated adaptive immunity, cytokine-driven inflammation, and FLS hyperplasia [169]. Within the context of this disease, lactate has been identified as a pivotal metabolic bridge linking immune activation to matrix destruction. The infiltration of immune cells and activated synovial cells results in a glycolytic storm, which in turn leads to the generation of substantial lactate. This lactate has been shown to induce widespread protein lactylation modifications, resulting in the trapping of inflammatory and matrix cells in a persistently activated pathological state at the epigenetic level. This has been demonstrated to be a significant contributing factor to the chronicity and intractability of the disease.

#### 3.3.1 Targeting lactate metabolism in RA

In contrast to the hypoxic, cell-autonomous glycolysis observed in OA cells, RA is characterized by a coordinated, cytokine-driven “glycolytic burst” that is initiated by infiltrating macrophages, T cells, and FLSs [110,170]. Clinical studies have indicated that serum lactate concentrations in patients suffering from RA are significantly elevated, reaching 2.6-3.7 times the levels found in healthy controls [171]. Elevated lactate levels in both serum and synovial fluid have been shown to correlate robustly with disease activity and radiographic progression [[171], [172], [173], [174]], and identified as a functional biomarker that aids in distinguishing RA from OA [175]. Mechanistically, the excess glycolysis is driven by the upregulation of key glycolytic enzymes and regulators such as GLUT1, HIF-1α, cellular myelocytomatosis oncogene (c-Myc), and PKM2 in macrophages and other synovial cells [176]. It has been demonstrated that this process is associated with the activation of the NLRP3/Casp-1/GSDMD pyroptosis pathway in synovial macrophages, thus leading to the amplification of inflammatory cell death [177]. Furthermore, elevated extracellular lactate directly stimulates the proliferation of RA synovial fibroblasts (RASFs), thereby contributing to the pathological synovial hyperplasia that is characteristic of RA [178].

Recently, therapeutic strategies that target this dysregulated signaling have attracted considerable interest. Interventions are multifaceted, focusing on key enzymatic nodes such as GLUT1, HK2, PKM2, PFKFB3, and LDH to directly inhibit lactate production [[111], [112], [113], [114], [115],^171^](Table 3). It has been demonstrated that alternative approaches can be employed to modulate upstream regulators, including eukaryotic elongation factor 2 kinase (eEF2K) and SET and MYND domain-containing protein 5 (SMYD5)-mediated Forkhead box protein O1 (FoxO1) methylation, with the objective of indirectly normalizing metabolic flux [116,179]. Pharmacological agents such as dimethyl fumarate have been shown to be efficacious in reducing lactate release and FLS proliferation [180,181]. Natural compounds, including Parishin E, a constituent of the orchid plant *Gastrodia elata* Bl., and geniposidic acid, a constituent of ginger-processed Eucommiae Cortex, have been shown to possess anti-RA properties, which are partly attributable to the repression of HK2 and LDHA expression. This effect has been demonstrated to impede glycolysis and subsequent macrophage polarization [182,183]. Furthermore, non-pharmacological approaches, including near-infrared photocatalytic hydrogen production, have demonstrated potential in experimental models through the elimination of excess lactate and the suppression of inflammation [117]. These diverse strategies emphasize the therapeutic potential of normalizing synovial metabolism in RA.

#### 3.3.2 Targeting lactate shuttle in RA

Dysregulated expression and function of MCTs have been demonstrated to be active contributors to the pathogenesis of RA, thus establishing a metabolic conduit that sustains inflammation and tissue damage. In FLS, the MCT4 plays a pivotal role in facilitating lactate export. Genetic silencing or pharmacological inhibition of *Mct4* has been shown to disrupt this metabolic balance, thereby triggering a series of events including, but not limited to, the induction of apoptosis, a reduction in glucose uptake and lactate production, the restoration of mitochondrial function and a reduction in oxidative stress. Collectively, these changes mitigate the severity of disease in preclinical models, such as collagen-induced arthritis (CIA), thereby highlighting the pivotal role of MCT4 in perpetuating the pathological synovial phenotype [118,119,184] (Table 3). Beyond the established MCT family, the SLC5A12 has emerged as a critical lactate sensor and importer in CD4^+^ T cells within the inflammatory niche. In this instance, lactate orchestrates a feed-forward loop, upregulating its own transporter, SLC5A12, on the T cell surface. This enhanced uptake has been demonstrated to promote T cell retention within inflamed tissue and drive pathogenic differentiation. Intracellular lactate activates the nuclear PKM2/STAT3 signaling axis and increases fatty acid synthesis (FAS), ultimately driving the production of the pro-inflammatory cytokine IL-17. The functional significance of this pathway has been confirmed by studies showing that antibody-mediated blockade of SLC5A12 ameliorates disease in murine arthritis models, thus nominating it as a precise immunometabolic checkpoint [120] (Table 3). Consequently, lactate transporters function as pivotal gatekeepers within the RA joint. The role of these proteins extends beyond facilitating passive metabolite flow; they also actively regulate cellular crosstalk by shaping lactate gradients. The presence of MCT4 in stromal cells and SLC5A12 in lymphocytes demonstrates the capacity for cell-type-specific transport mechanisms to underpin discrete pathological programmes. The observed findings are consistent with the diagnoses of synovial hyperplasia and inflammatory T cell responses. The targeting of these specific conduits is a promising therapeutic strategy for disrupting the metabolic communication that fuels self-perpetuating inflammatory loops in RA.

#### 3.3.3 Targeting lactylation modifications in RA

The clinical significance of lactylation in RA is underscored by the direct correlation between elevated plasma lactate levels, increased pan-lysine lactylation (Pan-Kla) in peripheral blood mononuclear cells, and greater clinical disease severity [185]. Specific LRGs, including IKAROS family zinc finger 1, lymphocyte cytosolic protein 1, Wiskott-Aldrich syndrome protein actin-nucleation promoting factor, NADH dehydrogenase (ubiquinone) 1 beta subcomplex subunit 3, N-Glycanase 1, and SLC25A4 have been identified as potential markers for early diagnosis and activity assessment [185,186]. At the tissue level, histone lactylation is significantly enriched at specific lysine residues, particularly H3K14, and H3K18, within RA-FLSs. These modifications act as epigenetic switches that promote the transcription of pro-inflammatory and matrix-degrading genes, thereby cementing the pathogenic, hyperproliferative phenotype of these cells [121,187]. A significant mechanistic exemplar is lactate-induced H3K18la, which has been demonstrated to promote RA-FLS proliferation and synovial hyperplasia by activating the methyltransferase 1 (METTL1)/NeuroD1/glutathione peroxidase 4 (GPX4) signaling axis, thereby conferring resistance to ferroptotic cell death [178]. This finding has been validated in CIA models. Conversely, the deacetylase SIRT3 exerts a protective effect by actively removing lactyl marks, specifically by de-lactylating H3K18la [121]. In consideration of its pathogenic role, therapeutic strategies targeting lactylation are logically focused on disrupting this epigenetic layer. The natural compound gastrodin (GAS) exemplifies the first strategy, ameliorating pathology in adjuvant-induced arthritis (AIA) models by inhibiting lactate production and H3K9la, leading to downregulation of key mediators like IL-6 and MMP-1/13 [122] (Table 3). Other natural agents, such as Parishin E and geniposidic acid, function partly by inhibiting H3K18la, H3K27la or H3K56la, respectively [182,183]. Recent research has expanded the therapeutic landscape by establishing a link between lactylation and RA-associated neuropathic pain. The natural flavonoid luteolin has been shown to attenuate chronic pain in CIA models by targeting a specific metabolic-epigenetic-transcriptional axis. Luteolin has been shown to bind directly to and inhibit LDHA, thereby reducing intracellular lactate and decreasing H3K9la levels on the promoter of NFATc2, which epigenetically silences its transcription. Downregulation of NFATc2 subsequently suppresses the differentiation and spinal infiltration of pro-inflammatory Th17 cells, a process critical for driving central sensitization and microglial activation associated with chronic pain [188]. This LDHA/H3K9la/NFATc2 axis elucidates a previously unobserved mechanism, revealing how targeting lactylation can address the neuroimmune component of RA and thereby complement existing strategies focused on synovial pathology.

Additionally, lactylation of cyclic GMP-AMP synthase (cGAS) has been demonstrated to impede its proteasomal degradation, resulting in its abnormal stabilization and constitutive activation of the downstream cGAS-stimulator of interferon genes (STING) pathway. This process has been shown to induce a state of macrophage-driven inflammation, thereby directly correlating the accumulation of lactate with the sustained activation of the innate immune system in patients diagnosed with RA [189]. The process of metabolic reprogramming within macrophages is subject to regulation by lactylation-dependent splicing events. Reduced expression of the splicing factor RNA binding motif protein 25 (RBM25) has been demonstrated to promote exon skipping in ATP-citrate lyase (ACLY) mRNA, resulting in the generation of a short isoform of ACLY. This, in turn, has been shown to enhance glycolytic flux, acetyl-CoA production, and a feed-forward loop of lactylation-dependent macrophage hyperactivation [190]. In RA FLS, lactylation has been observed to modify key functional proteins, including PKM2 and FTH1, thereby directly promoting pathogenesis. In particular, the process of artemisinin (ART)-facilitated lactylation of PKM2 at K166 has been shown to impede its nuclear translocation and downstream pro-inflammatory signaling [123] (Table 3). Furthermore, the level of FTH1 lactylation at K69 has been demonstrated to correlate with clinical disease activity, thereby underscoring its role as a biomarker that links cellular metabolism to systemic inflammation [191]. The findings, when considered collectively, suggest that lactylation functions as a pivotal regulatory mechanism, converting the local metabolic state into ongoing inflammation, metabolic dysfunction and synovial hyperplasia.

### 3.4 Lactate in the pathogenesis and therapy of IVDD

IVDD is a multifactorial pathological process characterized by dysregulated ECM metabolism, chronic low-grade inflammation, and accelerated cellular senescence, ultimately leading to structural failure and pain [192]. Lactate functions as the fundamental metabolic product that sustains NPCs during hypoxic metabolism. However, its pathological overaccumulation directly transforms into a metabolically toxic substance that acidifies the microenvironment and induces cellular stress-induced death. The degenerative process unfolds along the lactate production-acidosis axis, with acidosis itself becoming the dominant factor triggering programmed cell death. This mechanism is distinctly divergent from the inflammatory patterns observed in other vascularized tissues.

#### 3.4.1 Targeting lactate metabolism in IVDD

As previously outlined, within the healthy NP, baseline glycolysis and lactate production represent vital adaptations to hypoxia. In the process of degeneration, this homeostatic flux is amplified to a catastrophic degree. Pro-inflammatory cytokines, such as IL-1β, and sustained catabolic signaling are primary drivers of this glycolytic shift, often mediated through pathways like mTORC1 hyperactivation [125]. This transition has been demonstrated to result in the substantial accumulation of lactate, which has been observed across various species and has been positively correlated with the severity of disc degeneration [[193], [194], [195]]. The accumulation of lactate is an active pathogenic mediator. It has been demonstrated that this process directly contributes to disc degeneration by inducing the senescence of NPC and oxidative stress. This, in turn, is mechanistically linked to the dysregulation of key cellular pathways, including the Akt/p21/p27/cyclin D1 and Akt/Nrf2/heme oxygenase-1 (HO-1) pathways [126] (Table 4). In addition, lactate has been demonstrated to instigate a self-perpetuating, pathological feed-forward loop. The extracellular accumulation of lactate has been demonstrated to trigger a calcium influx, leading to the assembly of the NLRP3 inflammasome and subsequent activation of Casp-1 and IL-1β release [196]. Conversely, IL-1β, once secreted, has been shown to promote c-Myc nuclear translocation, leading to upregulation of glycolytic enzymes. This, in turn, results in further acceleration of lactate production and the establishment of a vicious glycolysis-pyroptosis cycle that exacerbates degeneration [196].

Disruption of mitochondrial quality control, exemplified by impaired BNIP3-mediated mitophagy, further perturbs this system by reducing lactate/H^+^ efflux and compromising energy metabolism, contributing to early degenerative changes [197]. The re-establishment of metabolic homeostasis is a strategy that shows great promise in terms of therapeutic applications. The promotion of mitophagy, for instance via the activator TJ0113, has been demonstrated to induce metabolic reprogramming that results in a substantial reduction in lactate production [198]. A comparable effect is observed with glutamine supplementation, which has been demonstrated to inhibit glycolysis, decrease lactate levels, and ameliorate NPC senescence by modulating AMPKα [199]. While LDHA-mediated glycolysis, which is regulated by the SIRT1/c-Myc axis, can support cellular energy demands under stress [127], its pathological overactivation is a key therapeutic target. The efficacy of direct inhibition of LDHA using siRNAs or 2-DG, in addition to innovative approaches that utilize lactate-scavenging biomaterials such as lactate oxidase (LOX)-loaded nanozymes (MS@MCL) or hydrogels (LMGDNPs), has been demonstrated to reduce lactate levels, mitigate oxidative stress, and promote NPC survival and matrix anabolism [25,128,129](Table 4). Recent advancements exemplify the therapeutic potential of a systems-level approach that concurrently targets the intertwined metabolic and inflammatory axes. A representative study developed a dual-strategy, environmentally responsive hydrogel system for IVDD treatment, which co-delivers siLDHA and the anti-inflammatory polyphenol epigallocatechin (EGC) via phenylboronic acid-functionalized nanoparticles. Upon injection into the degenerative disc, the hydrogel responds to the acidic and ROS-rich microenvironment, triggering sustained release. The EGC, once released, has been shown to inhibit the NF-κB/NLRP3 pathway, thereby suppressing IL-1β-driven inflammation. In addition, the co-delivery of siLDHA has been demonstrated to prevent pathological glycolysis and lactate overproduction. This coordinated intervention has been shown to disrupt the vicious inflammation-lactate cycle, alleviate lactate-induced ferroptosis in NPCs, and promote ECM synthesis and disc repair in vivo [200]. This integrated strategy emphasizes the transition from single-target interventions to sophisticated, multifunctional delivery platforms designed to normalize the dysregulated lactate metabolism central to IVDD progression.

#### 3.4.2 Targeting lactate shuttle in IVDD

The function of MCTs is to create and maintain the pathological lactate gradients that are observed in degeneration. The utilization of sophisticated in vitro models that replicate the physiological hypoxia and lactate gradients of the disc, including five-layered gelatin sponge systems, has been demonstrated to exhibit a synergistic amplification of catabolic signaling, encompassing the upregulation of MMP-3. It is noteworthy that the inhibition of MCT1 has been shown to reverse this effect, underscoring the active role of lactate shuttling in perpetuating degenerative signaling [130] (Table 4). Furthermore, lactate has been identified as a primary driver of extracellular acidification within the hypoxic disc niche. This results in an acidic microenvironment, which is sensed by a family of proton-gated ion channels. The most notable of these are acid-sensing ion channels 1a and 3 (ASIC1a and ASIC3), which have been found to be significantly upregulated on multiple cells such as endplate chondrocytes and NPCs in degenerated discs [[201], [202], [203], [204]]. The activation of these channels by extracellular protons and lactate itself instigates a detrimental signaling cascade. Upon activation, the ASICs facilitate a rapid influx of Ca^2+^, which functions as a pivotal second messenger, thereby elevating the intracellular levels of ROS, activating the NF-κB inflammatory pathways, promoting NLRP3 inflammasome assembly, and ultimately inducing the release of IL-1β [131,205,206]. This sequence of events directly contributes to the onset of key pathological cell fates in IVDD, including NPC pyroptosis, apoptosis, and senescence. The therapeutic relevance of this pathway has been demonstrated by studies that have shown that pharmacological inhibition of ASIC1a (e.g., with psalmotoxin 1) or modulation of associated pathways (e.g., the STAT3/SIRT3 axis by cryptotanshinone) can mitigate lactate/acidosis-induced cellular damage [205,207]. Furthermore, the acidic microenvironment has been demonstrated to sensitize other mechanosensitive channels, such as transient receptor potential vanilloid 4 (TRPV4), to mechanical stress [208]. The integration of metabolic and biomechanical stimuli has been shown to exacerbate disease progression. Collectively, these acid-responsive systems (i.e. ASICs, MCTs and TRPV4) comprise a coordinated sensory network that detects, amplifies, and translates the metabolic stress of lactate accumulation into downstream catabolic and inflammatory responses, making them integral components of the degenerative cascade.

#### 3.4.3 Targeting lactylation modifications in IVDD

As demonstrated by numerous studies, a positive correlation exists between the level of global lactylation and the progression of IVDD. A lactylation-related gene signature has been identified as a potential diagnostic tool, with Chromobox homolog 3 inhibition (e.g., by atosiban acetate) demonstrated to reduce glycolysis and global lactylation, thereby alleviating degeneration [132]. The metabolic basis for this elevated lactylation is underscored by the altered nutrient landscape in the degenerate disc. In human NP tissues that have undergone severe degeneration, and in NP tissues from aged rats, a decline in glutamine has been observed to coincide with increased lactate accumulation and lactylation [199]. The supplementation of glutamine has been demonstrated to counteract this shift by inhibiting glycolysis, reducing lactate production, and modulating AMPKα lactylation. This metabolic correction has been demonstrated to reduce NPC senescence and enhance protective autophagy and matrix synthesis [199]. Furthermore, lactate-driven histone lactylation, particularly at H3K18, has been shown to serve as a pivotal epigenetic switch, with context-dependent outcomes. In one pathogenic cascade, lactate-induced H3K18la and ACSL4 lactylation has been shown to activate ferroptosis and exacerbate ECM degradation [133] (Table 4). Conversely, the attenuation of metabolic stress has been demonstrated to reverse this process, thereby inducing a protective state. Activation of mitophagy (e.g., by TJ0113) has been demonstrated to reduce lactate levels and subsequent H3K18la, leading to the suppression of the pro-inflammatory gene thrombospondin-1 (THBS1). This establishes a protective lactate-H3K18la-THBS1 axis, demonstrating how modulating a specific lactylation mark can interrupt a key inflammatory pathway [198]. This finding provides a novel framework for identifying therapeutic targets that extend beyond the scope of metabolic enzymes themselves. Strategies that target the reduction of the lactate pool (via metabolic modulators such as glutamine or lactate-scavenging nanozymes) or, with greater precision, the inhibition of the specific writer enzymes responsible for the occurrence of pathogenic lactylation events (e.g. targeting p300/CBP in specific cell types) offer promising avenues to disrupt this deleterious signaling layer without globally impairing essential epigenetic regulation. Beyond the inhibition of writer enzymes, a more precise strategy involves the direct prevention of lactylation at a specific pathogenic site. A seminal study identified lactylation of SOD1 at lysine 123 (SOD1K123la) as a key event impairing antioxidant defence in NPCs. Utilizing virtual screening methodologies, a small molecule (ZL-01) was identified that binds specifically to this modification site, thereby acting as a direct inhibitor of SOD1K123la. Inhibition of SOD1K123la by ZL-01 resulted in the restoration of SOD1 activity, the alleviation of oxidative damage, and the attenuation of IVDD progression in vivo [209]. This development paves the way for more precise interventions in IVDD.

### 3.5 Lactate in the pathogenesis and therapy of bone malignancies

Bone malignancies, encompassing primary OS and bone metastases from carcinomas, represent a formidable therapeutic challenge due to their complex microenvironment and frequent treatment resistance [210,211]. In primary and metastatic bone malignancies, lactate is systematically hijacked by tumor cells and transformed into a multifunctional agent that supports their survival and expansion. The Warburg effect is a hallmark of tumor cells, which generate substantial lactate in order to fuel their own proliferation and to remodel the local microenvironment. This, in turn, facilitates immune evasion, bone tissue destruction and pain generation. In the context of bone metastases, tumor-derived lactate is utilized by OCs to accelerate bone resorption while simultaneously suppressing anti-tumor immunity. This underscores the intricate function of lactate in promoting tumor progression.

#### 3.5.1 Targeting lactate metabolism in bone malignancies

In primary and metastatic bone malignancies, reprogrammed cellular metabolism is a defining feature, with elevated lactate production serving as both a consequential biomarker and a functional driver of disease progression. Elevated serum LDH levels and increased intratumoral lactate concentration have been shown to be consistently associated with metastatic potential and reduced overall survival, thus establishing them as critical clinical prognostic indicators [134,[212], [213], [214]]. This metabolic phenotype is predominantly sustained by the Warburg effect, which is characterized by a reliance on aerobic glycolysis even in the presence of oxygen. The expression and activity of LDHA are pivotal to this process, as they facilitate tumor proliferation, invasion, and the acidification of the tumor microenvironment [135]. The regulation of LDHA expression is multifaceted, involving a complex, multi-layered network. Epigenetic modifiers, such as the lysine demethylase 6B (KDM6B), have been shown to promote LDHA transcription [136]. In addition, post-transcriptional mechanisms have been demonstrated to exert fine-tuned control. These include N-acetyltransferase 10 (NAT10)-mediated RNA acetylation influencing mRNA stability [137], as well as microRNAs (e.g., miR-323a-3p and miR-329-3p) and competitive endogenous circRNAs (e.g., circ_0000376 and circ-CNST) that sequester regulatory RNAs to modulate LDHA expression [[138], [139], [140], [141]]. A recently elucidated axis, the C1QTNF1-AS1/miR-346/PDK1-LDHA circuit, reveals a novel layer of metabolic regulation in OS. In this pathway, the long noncoding RNA C1QTNF1-AS1 co-activates the key glycolytic enzymes PDK1 and LDHA, whereas the miR-346 acts as a dual-target suppressor. Disrupting this axis has been shown to induce a synthetic co-metabolic crisis, which has been demonstrated to effectively reduce lactate output and sensitize tumors to chemotherapy. This, in turn, has been shown to expose a critical therapeutic vulnerability [215]. In addition to LDHA, the LDHB isoform has also been observed to be overexpressed in metastatic OS, with the potential to contribute to aggressive phenotypes. This finding underscores the broader significance of lactate metabolism in malignant contexts [216]. The targeting of these key glycolytic enzymes has become a core strategy for reversing the immunosuppressive tumor microenvironment. For instance, in advanced OS, the heat shock protein 90 (HSP90) inhibitor Ganetespib has been observed to concurrently reduce the expression of HK2 and PKM2, thereby effectively impeding aerobic glycolysis and substantially decreasing lactate production in tumor cells. The metabolic reprogramming that has been observed to alleviate local tumor acidosis and reduce global protein lysine lactylation [217]. Collectively, these changes have been demonstrated to remodel the immunosuppressive tumor microenvironment and enhance the response to immunotherapy.

Furthermore, the presence of lactate within the metastatic niche has been demonstrated to actively remodel the cellular environment. It has been demonstrated that this process promotes the expression of chemokines (e.g., CXCL10) and adhesion molecules (e.g., cadherin-11) in OC precursor cells via PI3K/Akt signaling, thereby fostering OC differentiation, recruitment, and the formation of a pro-fibrotic niche that exacerbates bone destruction [218,219]. In prostate cancer bone metastases, the E3 ligase tripartite motif-containing protein 28 enhances LDHA transcription through an autophosphorylation-dependent mechanism, amplifying glycolysis and lactate production to drive metastatic outgrowth [220]. Collectively, lactate functions as a central regulator that integrates tumor-intrinsic metabolic reprogramming with the manipulation of the bone microenvironment to fuel malignancy.

#### 3.5.2 Targeting lactate shuttle in bone malignancies

In bone metastases, lactate has been demonstrated to exert a pivotal regulatory influence on the intricate interactions between tumor cells and the surrounding stroma. The functional impact of this phenomenon is critically dependent on its dynamic shuttling via MCTs. Tumor cells, engaged in aerobic glycolysis, export lactate into the bone microenvironment via MCTs, particularly MCT4. The importation of this extracellular lactate by OCs is primarily facilitated by MCT1. The intercellular lactate shuttle has been demonstrated to fuel the oxidative metabolism of OCs, enhancing their resorptive capacity and directly facilitating the progression of osteolytic lesions. This process perpetuates a detrimental cycle of bone destruction, which in turn facilitates tumor growth [221]. The expression of MCT isoforms has been observed to be increased in cases of bone malignancies, and this has been shown to correlate with a poor prognosis. This underscores their role as both functional effectors and independent prognostic indicators. In OS, MCT1 and MCT4 have been shown to exhibit complementary yet distinct roles. Pharmacological inhibition of MCT1 has been demonstrated to impede tumor growth and metastasis, a process that may be facilitated by the disruption of intracellular pH homeostasis and the induction of oxidative stress [142,222]. MCT4 inhibition has been shown to reduce glycolytic dependency, glucose uptake, and proliferation [222]. Given their non-redundant functions in maintaining metabolic symbiosis within the tumor, combined inhibition of MCT1 and MCT4 presents a promising, synergistic therapeutic strategy for OS [221]. In addition to its role in fueling tumor-stromal metabolic coupling, MCT-mediated lactate transport also contributes to the manifestation of the debilitating symptoms associated with bone metastases. Lactate produced within the metastatic niche can be exported via MCT4 and subsequently taken up by sensory neurons in the dorsal root ganglia [223]. Consequently, MCTs cannot be regarded as passive conduits; rather, they function as active regulators that facilitate intratumoral metabolic symbiosis and OC-mediated bone destruction. The strategic inhibition of specific MCT isoforms is a rational approach to simultaneously disrupting tumor metabolism and halting osteolytic progression.

#### 3.5.3 Targeting lactate receptor in bone malignancies

In OS, the activation of GPR81 instigates a signaling cascade that exerts a direct detrimental effect on anti-tumor immunity and promotes invasiveness. Mechanistically, the recruitment of protein phosphatase 2A (PP2A) via β-arrestin2 by ligand-bound GPR81 results in the dephosphorylation and subsequent functional impairment of the tumor-suppressive transcription factors STAT1 and STAT2 [143]. It is evident that STAT1/2 play a pivotal role in the mediation of anti-proliferative responses. The inactivation of these proteins has been shown to facilitate immune evasion and enhance the invasive potential of OS cells. The pathological relevance of this axis is confirmed by the observation that pharmacological inhibition of PP2A with Endothall reverses these effects, suppressing OS cell proliferation and migration while inducing apoptosis. This establishes the lactate-GPR81-PP2A-STAT1/2 axis as a novel therapeutic target to counteract lactate-driven immune suppression in OS. Moreover, the available evidence indicates that GPR81 signaling has a crucial part to play in the pathogenesis of cancer-induced bone pain, a condition that significantly impacts patients with bone metastases. Lactate exported from metastatic lesions is transported to sensory neurons within the dorsal root ganglia, where it activates neuronal GPR81. The activation of this pathway has been demonstrated to initiate the ERK1/2-CREB pathway, induce calcium influx, and consequently result in mechanical hyperalgesia [223]. This mechanism positions lactate, via GPR81, as a “metabolite-neurotransmitter” that directly translates the metabolic activity of a distant tumor into nociceptive signaling within the nervous system. To summarize, GPR81 has been demonstrated to exert a dual pathological role in cases of bone malignancies. In the context of the tumor, it has been demonstrated that this process transduces a lactate signal, which suppresses anti-tumor immunity and promotes aggression. In contrast, within the sensory nervous system, GPR81 has been observed to convert the same metabolic signal into a potent pain stimulus. This dual function of GPR81 is indicative of its potential as a therapeutic target, offering the prospect of targeting both tumor progression and the debilitating symptoms associated with bone metastases.

#### 3.5.4 Targeting lactylation modification in bone malignancies

Lactylation facilitates the process by which tumors co-opt their own metabolic waste product to effect long-lasting transcriptional changes that favor survival and progression. A seminal example of this metabolic-epigenetic-immune axis is found in OS, where lactate induces H3K9la within tumor cells. It has been demonstrated that this modification has the capacity to promote the transcriptional upregulation of the gene which encodes the macrophage migration inhibitory factor (MIF). The secreted MIF exerts a paracrine effect on tumor-associated macrophages, thereby repressing their production of the T-cell chemoattractants. This suppression has been demonstrated to exert a deleterious effect on the recruitment and cytotoxic function of CD8^+^ T cells, thereby establishing an immunosuppressive niche that facilitates immune evasion and tumor progression [134]. The lactate-H3K9la-MIF axis has been demonstrated to provide a direct mechanistic link from glycolytic flux to the functional impairment of adaptive immunity. The broad impact of lactylation is further underscored by integrative multi-omics analyses, which have identified specific lactylation-related gene signatures strongly associated with poor prognosis in OS patients [22,134,224]. These signatures serve as significant biomarkers, facilitating risk stratification. Furthermore, they indicate core biological pathways that are hijacked by lactylation-driven epigenetic remodeling, thus offering insights into the molecular taxonomy of aggressive disease. In lung cancer bone metastasis, a parallel axis has been identified that centres on SLC2A3, a key player in driving glycolytic flux and lactate production. The accumulation of lactate has been shown to induce the lactylation of the tumor suppressor p53 at lysine 120 (p53K120la), thereby repressing its transcriptional activity and promoting tumor cell proliferation and metastasis. The SLC2A3/lactate/p53 lactylation axis has been demonstrated to promote OC differentiation and impair CD8^+^ T cell function. This integrated axis thus integrates tumor-intrinsic survival, bone remodeling and immune evasion into a comprehensive pathogenic mechanism [225]. Collectively, these findings establish lactylation as a master regulatory layer through which the high-lactate tumor microenvironment imposes durable changes on both cancer cells and immune constituents.

## 4 Limitations and future perspectives

This review outlines the regulatory role of lactate in maintaining bone homeostasis and in the pathogenesis of related diseases, and explores its potential therapeutic value. However, it should be noted that this field is undergoing rapid evolution, and as a result, numerous questions remain unanswered. Consequently, the identification of future research directions is imperative for the translation of basic research findings into clinical applications.

### 4.1 Context-dependent functions of lactate

Recent studies have highlighted a fundamental paradox in the function of lactate. Within the context of physiological conditions, the primary function of lactate is to facilitate intercellular energy metabolism and act as an anabolic signaling molecule. However, in pathological microenvironments, such as those associated with OS or joint inflammation, the excessive accumulation of lactate can exacerbate disease progression through interactions between metabolic and epigenetic mechanisms. This seemingly contradictory dual role is rooted in the profound context-dependence of lactate's biological effects, which is also the root cause of the numerous controversies currently surrounding this field. This concentration-dependency is vividly illustrated across different contexts. In OB-lineage cells, lower lactate concentrations (e.g., 2.5-10 mM) have been observed to promote ALP activity and osteogenic gene expression, while higher concentrations (e.g., 20 mM) have been shown to potentially inhibit proliferation and induce apoptosis, thereby delineating a biphasic effect [40]. In OA, the transition from a potential metabolic substrate to a pathogenic driver occurs at a boundary estimated to be between 5 and 10 mM. Concentrations at or above 10 mM have been shown to induce injury to the cells via the GPR81/PI3K/NOX4/ROS axis [23] and to promote ferroptosis and fibrosis through histone lactylation (e.g., H3K18la)-mediated gene activation [104,106]. In RA, analogous to the aforementioned conditions, elevated lactate concentrations (approximately 10 mM) have been demonstrated to play a pivotal role in the activation of pathogenic programs. This process involves the induction of pro-inflammatory histone lactylation (e.g., H3K9la and H3K18la) in synovial fibroblasts, resulting in the enhancement of their inflammatory and invasive characteristics [121,122,124]. Additionally, these elevated lactate concentrations have been shown to promote the reprogramming of CD4^+^ T cell metabolism, thereby stimulating IL-17 production [120]. In IVDD, the pathological accumulation of lactate (5-20 mM in vitro) has been shown to drive NPC dysfunction through multiple mechanisms, including lactylation-induced ferroptosis [25], senescence [199], and pyroptosis [131]. It is noteworthy that in human discs, the baseline lactate concentration in the nucleus pulposus is approximately 2-6 mM, but can escalate to 12-16 mM in severe degeneration, thereby underscoring the in vivo relevance of this pathological threshold [226]. These examples underscore that the functional output of lactate is not arbitrary, but is gated by specific concentration windows that are disease- and cell type-dependent.

The aforementioned controversies and uncertainties stem from complex factors at multiple levels. From the perspective of intracellular properties, it is evident that skeletal cells of varying types and differentiation states, including OBs, OCs and chondrocytes, exhibit distinctly disparate responses to a uniform lactate stimulus. This phenomenon can be attributed to the inherent variations in their metabolic characteristics and receptor expression profiles. When considering the external microenvironment, the local oxygen partial pressure, the specific inflammatory cytokine profiles, the mechanical stimuli, and the concomitant existence of other metabolites, collectively these constitute the specific biological context for interpreting lactate signaling. At the methodological level, the heterogeneity of research models and experimental systems is a direct contributing factor. The two-dimensional cell culture systems currently employed are incapable of replicating the key in vivo characteristics of the skeletal system, including complex intercellular communication, dynamic metabolic microenvironments, mechanical stimuli, and immune cell infiltration. A more profound challenge is that extant studies often neglect the spatiotemporal dynamics and compartmentalized distribution of lactate within complex bone tissue; measurements of average concentrations from tissue homogenates fail to reflect the local micro-concentration gradients that determine cell fate [82]. Concurrently, a significant number of studies examine individual lactate pathways in isolation rather than conducting an integrated analysis within the broader context of cellular energy metabolism and stress responses. Indeed, the biological effects of lactate are closely linked to the homeostasis state of core energy and stress signaling pathways, such as HIF-1α activity, AMPK and mTORC1 [75]. From a metabolic perspective, the dynamic homeostasis between lactate and pyruvate is of pivotal significance in terms of regulatory processes. The lactate/pyruvate ratio is a core indicator of the intracellular NAD^+^/NADH redox state, whose abnormalities not only indicate an imbalance between glycolytic flux and OXPHOS, but also indirectly regulate various signaling pathways, including the stability of HIF-1α, by influencing the activity of NAD^+^-dependent enzymes such as sirtuin family deacetylases, and the levels of metabolites such as α-KG. This profound role in the final determination of OB fate is of particular significance.

In order to resolve existing controversies and deepen our understanding of the mechanisms involved, future research must advance in a coordinated manner from both methodological and scientific perspectives. The development of in vitro experimental systems capable of more accurately simulating the complex in vivo microenvironment is of crucial importance. Such systems could, for example, be established by means of three-dimensional co-culture systems or organoids that incorporate diverse cell types, such as bone, cartilage, immune, and vascular endothelial cells. The integration of mechanical loading with dynamic perfusion conditions is a key feature of these systems. The application of advanced technologies with high spatio-temporal resolution is fundamental to enable in situ, real-time monitoring of lactate distribution, flux and kinetics within the bone microenvironment. The underlying rationale for this phenomenon is that it facilitates the establishment of scientific definitions for the concentration thresholds associated with its physiological and pathological functions. At the single-cell or spatio-omics level, integrated analysis of the relationship between lactate metabolic status and global molecular phenotypes (transcriptome, proteome, epigenome) will facilitate elucidation of cell-type-specific features of lactate regulation. It is imperative that future research moves beyond the description of isolated molecular events and instead focuses on the systematic elucidation of how lactate interacts with core cell fate-determining signaling pathways (e.g. Wnt/β-catenin, RANKL/OPG, TGF-β/BMP) under different pathophysiological conditions. This will be essential for a comprehensive understanding of the principles underlying its context-dependent functions.

### 4.2 Regulatory nodes and targeted interventions in lactate signaling

The ultimate biological effects of lactate are determined by multiple factors, with its specific functional outcomes highly dependent on which downstream signaling pathways are engaged. Receptor-mediated cascades represent the fundamental initial step in determining the direction of these effects. Activation of the GPR81 typically mediates anti-inflammatory and anabolic responses [41,42], whereas in specific pathological contexts, lactate can trigger pro-inflammatory and catabolic signals via alternative receptors such as ASICs or through MCTs [131,227]. It is noteworthy that GPR81 signaling may exhibit a bias that is contingent upon both ligand concentration and conformation, resulting in divergent biological outcomes. However, further elucidation is required to determine the specific manifestations and regulatory mechanisms of this bias in skeletal cells [96].

At the level of transcriptional and post-transcriptional regulation, a core mechanism of lactate's action involves the post-translational modifications it induces, particularly histone lactylation. The dynamic establishment and removal of histone lactylation constitute a key layer of epigenetic regulation, the output of which exhibits high genomic site specificity and context-dependence [105,121]. However, given the inherent complexity of lactylation, including its site-specificity, dynamic reversibility and crosstalk with other modifications (e.g. acetylation and methylation), a major challenge arises in elucidating its precise molecular mechanisms [25,106]. For instance, the processes of lactylation and acetylation may compete for the same lysine residue (e.g., at H3K18), thereby facilitating opposing effects on gene transcription. Furthermore, lactylation has been demonstrated to function as an upstream signal regulating other epigenetic layers. In OBs, fluctuations in H3K18la levels, driven by lactate metabolism, regulate the expression of the KH-type splicing regulatory protein (KSRP), which in turn influences the alternative splicing of downstream pro-fibrotic genes [228]. During the process of ligament ossification, a specific accumulation of H3K18la is observed in the promoter region of the Methyltransferase-like 3 (METTL3) gene. This accumulation activates the transcription of the METTL3 gene, leading to the methylation of m6A in the promoter regions of downstream osteogenic genes, such as BMP2. This process establishes a regulatory cascade that contributes to the differentiation and maturation of osteogenic cells [229]. In the process of dentin formation, the production of lactate is promoted by the KDM6B-PDK1 axis, leading to the lactylation of Zinc Finger E-Box Binding Homeobox 2 (ZEB2). This, in turn, regulates its transcriptional activity, thereby driving the process of cellular mineralization [230]. Collectively, these findings indicate a multi-tiered, interactive regulatory system extending from lactate to histone lactylation, and further to RNA modification or transcription factor function, underscoring the pivotal role of lactate in epigenetic regulation.

In accordance with the regulatory layers delineated above, prospective therapeutic interventions can be conceptualized at multiple levels. At the receptor/transporter level, the development of highly selective agonists or antagonists for targets such as GPR81 or ASICs represents a potential strategy for the correction of pathological signaling. At the epigenetic level, the targeting of the dynamic processes of lactate-mediated modifications may enable precise regulation of disease-associated gene expression. This objective could be realized through the development of tools to modulate the activity of 'writer' or 'eraser' enzymes, or by intervening in downstream effectors (e.g., METTL3 and ZEB2). Furthermore, the complexity of the lactate signaling network suggests that combination therapies, such as the simultaneous targeting of lactate metabolism in conjunction with existing immunomodulatory or bone-modulating agents, may enhance therapeutic efficacy.

### 4.3 Clinical translation pathways and challenges for targeting the lactate pathway

The translation of fundamental research in the field of lactate biology into clinically valuable interventions offers both opportunities and systemic challenges. The attainment of success is predicated on the progression of two concomitant pathways: the development of diagnostic biomarkers and the innovation of therapeutic interventions. The primary objective is to establish a non-invasive, dynamic monitoring system. The system is to be based on lactate metabolic flux or its specific modification products. The development of such a system necessitates a series of rigorous translational steps, commencing with the identification and validation of biomarkers in large prospective cohorts. The process employs a range of techniques, including metabolomics, proteolactylomics and hyperpolarized 13C-pyruvate magnetic resonance imaging. In order to achieve routine clinical application, there is a necessity for subsequent translation into standardized, high-throughput methods. It is imperative that trials guided by clinical hard endpoints confirm the added value of these biomarkers over existing standards for early warning, precise stratification, treatment response monitoring, and prognostic assessment. This will ultimately provide new tools for clinical decision-making.

With regard to therapeutic interventions, strategies that target key nodes such as LDH, MCTs, GPR81, and lactylation face fundamental challenges, primarily concerning target specificity and the risk of off-target effects. It has been hypothesized that LDH inhibitors may compromise normal function in highly glycolytic tissues, such as skeletal muscle and brain tissue [231,232]. Systemically administered MCT inhibitors have been demonstrated to disrupt whole-body metabolic homeostasis [233,234], while non-specific GPR81 antagonism has been shown to result in a range of unpredictable side effects [41]. The regulation of lactylation is a complex process. One approach to regulating lactylation is to target the catalytic enzymes that facilitate it, such as p300/CBP. However, this method can be problematic as it often lacks specificity. An alternative method is to regulate the substrate lactate levels indirectly, but this can disrupt numerous lactate-dependent physiological processes [108,235]. Consequently, the development of precise technologies with cell-type and disease-stage specificity is imperative to overcome this translational bottleneck.

Beyond pharmacological hurdles, disease heterogeneity, metabolic pathway compensation, and preclinical model limitations pose deeper systemic barriers. The heterogeneity of skeletal diseases necessitates the implementation of future clinical trials that will enable the precise stratification of patients using molecular markers (e.g. imaging, metabolic parameters, liquid biopsy lactate profiles) and the employment of adaptive trial designs. The compensatory capacity of metabolic pathways means that the inhibition of a single target (e.g. MCT1) may result in the upregulation of alternatives (e.g. MCT4), emphasizing the necessity for multi-target combinations or the pairing of novel lactate-targeting drugs with standard therapies to overcome resistance. Furthermore, extant animal models are inadequate in simulating the protracted natural history of human skeletal diseases or their complex tissue microenvironment, thus emphasizing the urgent requirement for more physiologically relevant humanized systems, such as bone organoids derived from patient iPSCs.

In order to address these challenges in a systematic manner, future translational research should focus on complementary strategic directions. Firstly, it is imperative to prioritize the clinical translation of advanced in vivo monitoring technologies and intelligent delivery systems. Standardizing the clinical applications of metabolic imaging (e.g., hyperpolarized MRI) is pivotal. Concurrently, the engineering of intelligent biomaterials that dynamically respond to local lactate or pH fluctuations at lesion sites is imperative. For instance, the design of bone-targeting nanoparticles encapsulating siRNA against glycolytic enzymes is required, as well as the development of pH-responsive hydrogels for on-demand release of lactate-clearing enzymes. As demonstrated by Qian et al. [163], research provides a proof-of-concept for a hydrogel-guided p-LOX nanodelivery system that achieved sustained drug release and deep penetration in an OA model. This system promoted cartilage repair via lactate metabolism modulation. Secondly, exploring diverse intervention modalities and initiating proof-of-concept clinical studies is critical. It is imperative that strategies encompass beyond traditional pharmacological interventions. It is evident that microbial interventions which target the gut-bone axis, through the production of bioactive substances, show unique potential for both the prevention and treatment of various diseases. Recent studies have indicated that certain lactic acid bacteria (e.g., Lacticaseibacillus rhamnosus) and their fermentation products have the capacity to inhibit OC differentiation/activation and reduce bone resorption in periodontitis [236]. It is noteworthy that bacterial fermentation has the capacity to catalyze the biotransformation of food proteins, thereby enhancing their anti-osteoporotic efficacy. A significant increase in isoflavones (e.g., genistein and daidzein) has been observed as a result of soy protein fermentation, with these isoflavones contributing to enhanced osteogenic gene expression and the inhibition of OC formation [237]. In addition, postbiotics derived from the fermentation of whey and grape seeds by lactic acid bacteria have been observed to enhance the expression of genes associated with muscle growth, anti-inflammation, and positive bone regulation in models of muscle atrophy [238]. The notion that these beneficial effects are mediated through dual mechanisms warrants further investigation. Firstly, it is important to note that probiotic therapies have the capacity to modulate systemic and local lactate levels, albeit indirectly. Secondly, and more directly, microbially derived bioactive metabolites such as specific short-chain fatty acids (SCFAs), polyphenol derivatives and bioactive peptides can reach the bone microenvironment via the circulation. Once in the bone microenvironment, these metabolites can interact with the local lactate signaling network. It is noteworthy that SCFAs, such as butyrate and propionate, are predominantly transported into cells via MCTs [239]. These MCTs belong to the same family that is responsible for lactate shuttling. This shared transport system creates a potential point of interaction (or interface) for crosstalk, where the influx of microbial metabolites could influence the intracellular lactate pool and its downstream signaling. Although direct experimental evidence for functional interaction between SCFA receptors (e.g., GPR41/43) and the lactate receptor (GPR81) in bone is currently limited, their co-expression on relevant bone and immune cells supports a plausible model of receptor-level crosstalk that merits future investigation. Thus, by engaging with the core transport (MCTs) and sensing (GPCRs) machinery of the lactate microenvironment, gut-derived signals could be directly integrated into local bone metabolic and immune responses. It is imperative to initiate proof-of-concept trials for both existing lead compounds and novel delivery systems in order to assess safety and early efficacy signals. This is a pivotal step towards the translation of lactate-targeted therapies from the laboratory to clinical practice.

## 5 Conclusions

Conventionally regarded as a by-product of metabolism, lactate is now recognized by the evidence synthesized in this review as a pivotal and intricate metabolic center within the skeletal system, executing a dual function in maintaining homeostasis and propelling disease progression. Within the context of physiological conditions, lactate fulfils the role of an intercellular energy carrier and an anabolic signal. This contributes to the delicate balance of bone formation, resorption, and cartilage metabolism. However, pathological accumulation of lactate has been observed to exacerbate a number of pathologies, including bone loss, cartilage destruction, synovial inflammation, and tumor progression across multiple tissue types under conditions such as OP, OA, RA, IVDD, and OS. The process is facilitated by the activation of specific receptor signaling pathways, the induction of pro-inflammatory and catabolic gene expression, and the promotion of diverse protein lactylation modifications. This paradoxical duality can be attributed to the profound context-dependence of lactate's actions. The ultimate functional output of this process is determined by a number of factors, including the integration of local lactate concentration, the duration of exposure, the identity and state of target cells, and the co-existing signaling milieu. This context-dependence provides a rationale for the inconsistencies observed in current research, and its comprehensive elucidation forms the scientific basis for developing effective therapies.

The intricate contextual regulation, as underscored by systemic barriers such as disease heterogeneity and metabolic pathway compensation, forms the foundation of the present bottlenecks in clinical translation. These include a limited understanding of in vivo lactate kinetics, the inability of current models to recapitulate human disease complexity, and the risk of off-target effects due to the ubiquitous expression of target molecules. Consequently, future research endeavors must transition from isolated molecular explorations to integrated, systems-based approaches. This involves the development of complex experimental systems that more accurately replicate pathophysiological conditions, the utilization of high spatio-temporal resolution techniques to delineate lactate metabolic heterogeneity within bone, the mapping of disease-specific multi-omics profiles to identify targets and biomarkers, and the subsequent design of cell- and disease-stage-specific precision strategies. To summarize, it is evident that lactate metabolism represents a pivotal pathway that is integral to the interconnectivity between cellular energy status, epigenetic remodeling, cell fate and tissue homeostasis in bone. Research into its roles has profoundly advanced our understanding of skeletal physiology and pathology, simultaneously revealing novel therapeutic avenues via targeting the local metabolic microenvironment.

## CRediT authorship contribution statement

Boyi Zong: Conceptualization, Writing - original draft, Writing - review & editing. Fengzhi Yu: Conceptualization, Writing - original draft, Writing - review & editing. Shichang Li: Supervision, Writing - review & editing. Peng Sun: Conceptualization, Funding acquisition, Writing - review & editing. All authors have read and approved the final manuscript and agree to its publication in the journal.

## Declaration of generative AI in scientific writing statement

No generative artificial intelligence (AI) or AI-assisted technologies were used in the preparation of this manuscript.

## Funding

This work was supported by grants from the National Natural Science Foundation of China (32171130 and 32571318).

## **Declaration of competing interest**

The authors declare that the research was conducted in the absence of any commercial or financial relationships that could be construed as a potential conflict of interest.
